# Loss of the androgen receptor suppresses intrarenal calcium oxalate crystals deposition via altering macrophage recruitment/M2 polarization with change of the miR-185-5p/CSF-1 signals

**DOI:** 10.1038/s41419-019-1358-y

**Published:** 2019-03-20

**Authors:** Wei Zhu, Zhijian Zhao, Fuju Chou, Li Zuo, Tongzu Liu, Shuyuan Yeh, David Bushinsky, Guohua Zeng, Chawnshang Chang

**Affiliations:** 1 Department of Urology and Guangdong Key Laboratory of Urology, The First Affiliated Hospital of Guangzhou Medical University, 510230 Guangzhou, China; 2 George Whipple Lab for Cancer Research, Departments of Pathology, Urology, and Radiation Oncology, and The Wilmot Cancer Institute, University of Rochester Medical Center, Rochester, NY 14646 USA; 3 Departments of Medicine, University of Rochester Medical Center, Rochester, NY 14646 USA; 4 Sex Hormone Research Center, China Medical University/Hospital, Taichung, 404 Taiwan

## Abstract

Crystals can trigger a wide range of kidney injuries that may link to the development of kidney stones. Infiltrating macrophages may influence hyperoxaluria-induced intrarenal calcium oxalate (CaOx) crystals deposition, yet their linkage to sex hormones remains unclear. Here we demonstrated that suppressing the androgen receptor (AR) expression in renal tubular epithelial cells increased the macrophage recruitment/M2 polarization that may result in enhancing the phagocytosis of intrarenal CaOx crystals. Mechanism dissection suggested that AR can suppress macrophage colony-stimulating factor 1 (CSF-1) expression via increasing miRNA-185-5p expression to suppress the M2 macrophage polarization-mediated intrarenal CaOx crystals phagocytosis. The preclinical study using glyoxylate-induced intrarenal CaOx crystals deposition mouse model revealed that renal tubule-specific AR knockout mice have less intrarenal CaOx crystals deposition with more recruited M2 macrophages in the kidney compared with the wild-type mice. Results from the in vivo rat model using hydroxy-l-proline-induced CaOx crystals deposition also demonstrated that targeting the AR with ASC-J9® suppressed the intrarenal CaOx crystals deposition via increasing the renal macrophage recruitment/M2 polarization. Together, results from multiple preclinical studies using multiple in vitro cell lines and in vivo mouse/rat models all demonstrated that targeting the AR with a small molecule ASC-J9® may function via altering macrophage recruitment/M2 polarization to decrease the intrarenal CaOx crystals deposition, a key phenotype seen in many kidney stone disease patients with hyperoxaluria.

## Introduction

Kidney stone disease is a common urological disease that poses a significant health care burden^1^. Calcium phosphate (CaP) wrapped/coated with calcium oxalate (CaOx) crystals account for approximately 70% of kidney stones, which are often associated with hypercalciuria and/or hyperoxaluria^2^. As the CaOx is slightly soluble, increases in urinary Ca^2+^ and/or oxalate concentrations under some selective conditions, may lead to increase the intrarenal CaP–CaOx crystals deposition on the renal parenchyma^3–5^, a key phenotype seen in hyperoxaluria-related kidney stone diseases.

Several in vitro^4–6^ and in vivo^7^ studies indicated that the CaOx crystals deposition and subsequent elimination could be altered by infiltrating macrophages, which are functionally classified into two types, pro-inflammatory M1 and anti-inflammatory M2 macrophages. M2 macrophages can directly suppress CaOx crystals deposition by phagocytizing crystals, whereas M1 macrophages may promote CaOx crystals deposition through altering the inflammation-related oxidative stress^5,8^.

Males have a higher kidney stone incidence compared with females, indicating that sex hormones may function through their receptors to influence kidney stone disease^9–11^. Early studies indicated that the expression of the androgen receptor (AR) was higher in male patients with kidney stone disease^12,13^, and Liang et al.^12^ also found AR signaling could promote CaOx crystals deposition via increasing liver oxalate biosynthesis and renal oxidative stress. The impact of AR signaling through altering the infiltrating macrophages to influence the intrarenal CaOx crystals deposition and elimination, however, remains unclear.

Here we used in vitro assays plus the in vivo mouse and rat model to demonstrate that targeting AR in renal tubular cells could promote the M2 macrophages recruitment/polarization to increase their phagocytic ability to decrease/eliminate the CaOx crystals deposition.

## Results

### Targeting AR in renal tubular epithelial cells increased the recruitment of macrophages

**Fig. 1:**
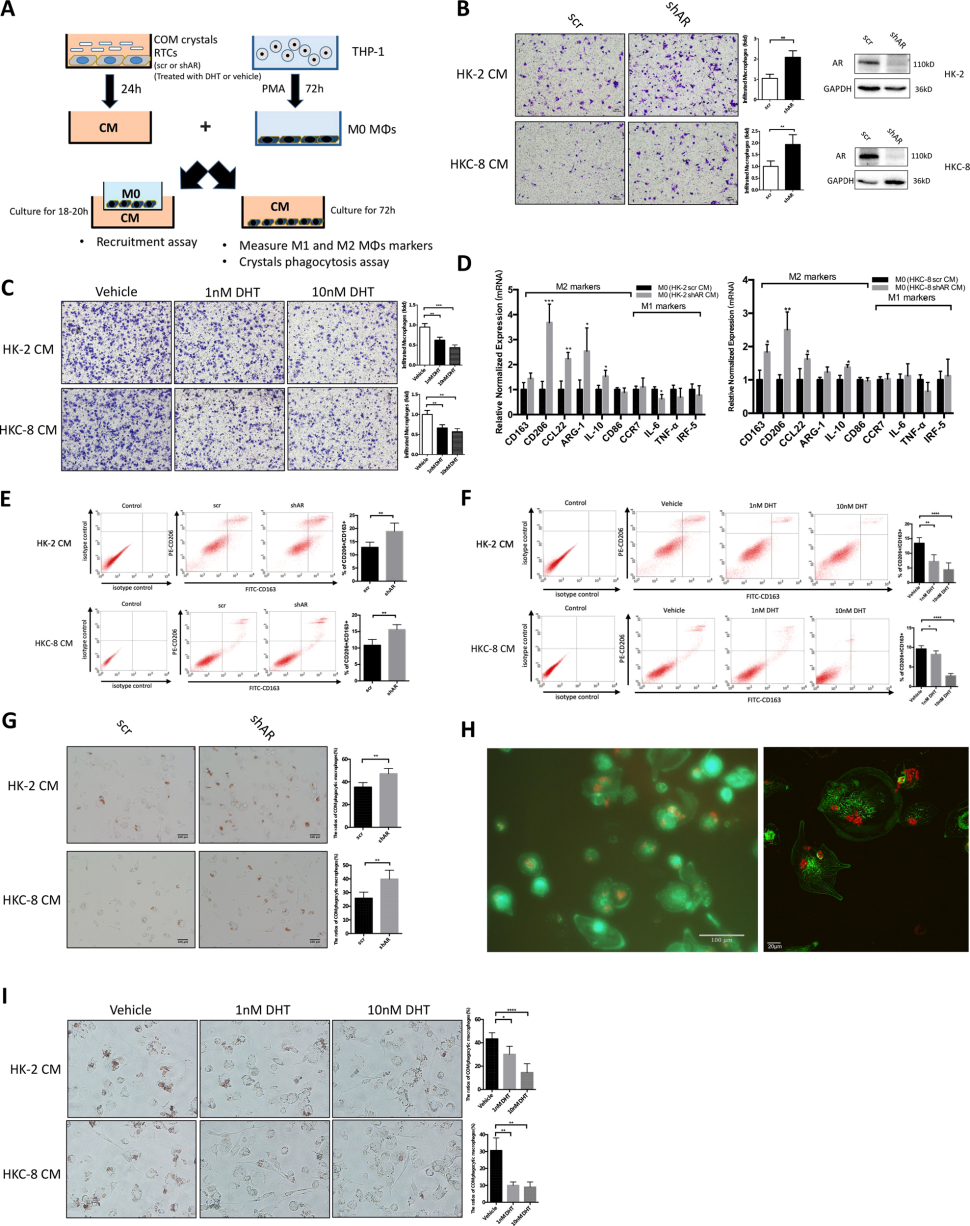
Targeting androgen receptor (AR) with shRNA in renal tubular epithelial cells (RTCs) increased the recruitment of macrophages, promoted macrophages M2 polarization, and increased calcium oxalate monohydrate (COM) crystals phagocytic ability of macrophages. **a** Expeimental outline for macrophage migration assay and crystals phagocytosis assay. Human THP-1 cells were differentiated into unpolarized macrophages (M0-MΦs) by incubation with 100 ng/ml PMA for 72 h. The conditioned media (CM) were collected from RTCs-scr/shAR (HK-2 or HKC-8 cells) treated with 20 μg/cm^2^ COM for 24 h, or RTCs treated with varying concentration of DHT and 20 μg/cm^2^ COM for 24 h. For macrophage migration (recruitment) assay, 1 × 10^5^/well M0-MΦs were added in the upper chambers, and the CM were placed into the lower chambers of transwell plates. For M1 and M2 markers and crystals phagocytosis assay, the M0-MΦs were incubated in CM that was diluted with 10% heat-inactivated serum RPMI media at 1:1 for 3 days. **b** Macrophage migration to the RTCs CM. The M0-MΦs migration to the RTCs HK-2 (scramble/shAR) CM and to the HKC-8 (scramble/shAR) CM were shown. Western blot right panels show AR knockdown efficiency. **c** Macrophage migration to the CM from RTCs treated with varying concentration of DHT and 20 μg/cm^2^ COM. **d** CM from AR-depleted RTCs showed increased mRNA levels of markers of M2 phenotypic MΦs, including CD163, CD206, CCL22, Arg-1, and IL-10 in MO-MØs after 72 h of incubation compared with CM from control (scramble) RTCs by quantitative real-time PCR (qRT-PCR). **e** Representative flow cytometry analysis of CD163 and CD206 expression in M0-MΦs after 72 h of incubation in CM from AR-depleted or control RTCs. **f** Representative flow cytometry analysis of CD163 and CD206 expression in M0-MΦs after 72 h of incubation in CM from RTCs treated with varying concentration of DHT. **g** Analysis of the COM crystals phagocytosis ability of MΦs after incubation with HK-2 (scramble/shAR) CM or HKC-8 (scramble/shAR) CM for 72 h. After 3 days incubation with CMs, the MΦs were treated with 15 μg/cm^2^ Ponceau-S-stained COM crystals. At 24 h, the crystals MΦs uptake was evaluated by optical microscopy. Representative images are shown (left panel). Quantification at right shows mean ± SD percent of MΦs containing phagocytized red material (right panel). **h** Fluorescence micrograph (left panel) and confocal laser scanning micrograph (right panel) of PMA-differentiated THP-1 MΦs phagocytosed COM crystals. The MΦs were stained by Cytopainter Phalloidin-iFlour 488 (green), and the COM crystals were stained by Ponceau-S (red). **i** Analysis of the COM crystals phagocytosis ability of MΦs after incubation for 72 h with CM from RTCs treated with varying concentration of DHT. For **b**, **c**, **e**, **f**, **g**, and **i** quantifications are at the right. All quantitations are presented as mean ± SD, *P < 0.05, **P < 0.01, ***P < 0.001, ****P < 0.0001 by Student’s *t*-test (**b, e**, **g**) or One-way ANOVA followed by Bonferroni multiple comparison test (**c, f**, **i**)

As recent studies indicate that macrophages may alter the intrarenal CaOx crystals elimination^14^, we were interested in testing the impact of the AR on the recruitment of macrophages to renal epithelial cells. We knocked-down AR with AR-shRNA in renal epithelial HK-2 cells and examined the impact on the phorbol 12-myristate 13-acetate (PMA)-induced THP-1 macrophages (M0-MΦs) recruitment to the renal epithelial HK-2 cells (see outline in Fig. 1a). The results revealed that knocking down AR increased the HK-2 cells conditioned media (CM) capacity to better recruit the M0-MΦs using the transwell migration system (Fig. 1b). Similar results were observed when we replaced the HK-2 cells with another renal epithelial cell line, HKC-8 (Fig. 1b), as well as replaced the THP-1 macrophages/HK-2 cells with mouse RAW264.7 macrophages/cortical collecting duct M1 cells (Supplementary Fig. 1A-B). Importantly, we also obtained similar results when we used another AR-shRNA to knockdown AR (Supplementary Fig. 2A-C). Conversely, adding 1 or 10 nM dihydrotestosterone (DHT) in HK-2 and HKc-8 cells resulted in decreased CM capacity to recruit the M0-MΦs (Fig. 1c).

### Targeting AR in RTCs promoted the infiltrated macrophage M2 polarization

To further study if targeting AR in renal epithelial cells can also influence the polarization of M0-MΦs into either inflammatory macrophages (named as M1-MΦs) or anti-inflammatory macrophages (named as M2-MΦs), we assayed the CM from renal epithelial cells with or without knocked-down AR for the impact on macrophage polarization.

As shown in Fig. 1d, we found knocking down AR in renal epithelial cells increased the polarization of M0-MΦs to M2-MΦs, with little influence on the M0-MΦs to M1-MΦs. Similar results were also observed when we replaced the THP-1 macrophages/HK-2 cells with RAW264.7 macrophages/M1 cells (Supplementary Fig. 1C). Results from flow cytometry also showed that M0-MΦs cultured in CM from renal epithelial cells with knocked-down AR had a higher percentage of M2-MΦs expressing CD163 and CD206 than the control cells (Fig. 1e), and adding 1 or 10 nM DHT in renal epithelial cells decreased the polarization of M0-MΦs to M2-MΦs (Fig. 1f).

Together, results from Fig. 1a-f and Supplementary Fig. 1A-C and 2A-C, demonstrate that targeting AR in RTCs increased the recruitment of macrophages and polarization of recruited macrophages to the M2 type.

### Targeting AR in RTCs enhanced the phagocytosis of CaOx crystals

To further study the impact of AR-modulated macrophages recruitment and polarization on the CaOx monohydrate (COM) crystals deposition, we then assayed the AR-mediated phagocytic ability of MΦs. As shown in Fig. 1g–h, MΦs cultured in the CM from renal epithelial cells with knocked-down AR had a higher rate of COM crystals phagocytosis than the control cells. Similar results were also observed when we used another AR-shRNA (Supplementary Fig. 2D), as well as replaced the THP-1 macrophages/HK-2 cells with RAW264.7 macrophages/M1 cells (Supplementary Fig. 1D). Conversely, MΦs cultured in the CM from renal epithelial cells treated with 1 or 10 nM DHT had a lower rate of COM crystals phagocytosis than the control cells (Fig. 1i).

Together, results from Fig. 1a-i and Supplementary Fig. 1A-D and 2A-D suggest that knocking down AR in renal epithelial cells increase M0-MΦs recruitment and promote the polarization of macrophages from M0-MΦs to M2-MΦs, which may then increase the ability of MΦs in phagocytosis of COM crystals.

### Mechanism dissection of how AR can alter the MΦs recruitment and M2-MΦs polarization: *via* up-regulating the macrophage colony-stimulating factor 1 (CSF-1) expression

**Fig. 2:**
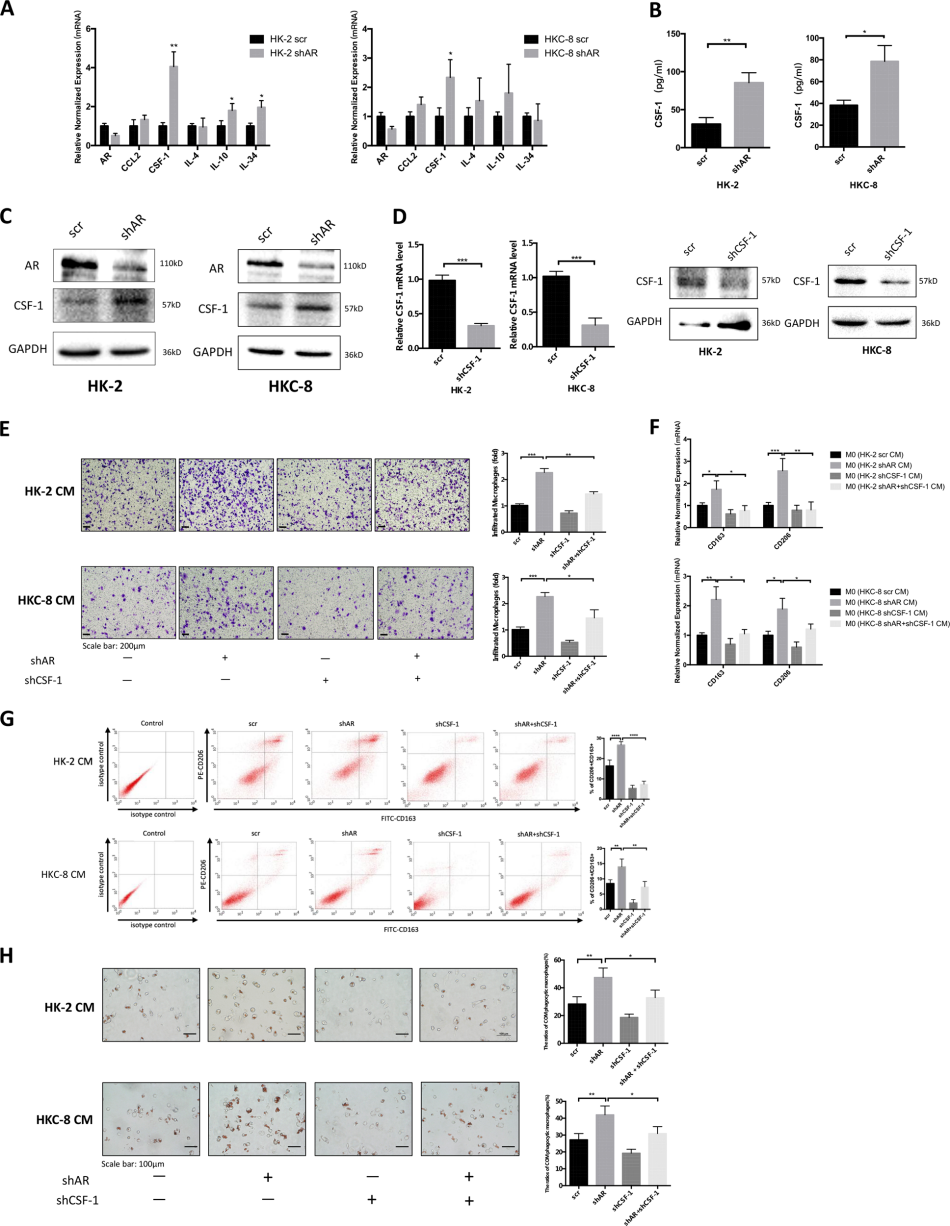
Knocking down androgen receptor (AR) in renal tubular epithelial cells (RTCs) enhanced the macrophages migration and M2 polarization via modulating the CSF-1 signals. **a-c** Cells were pre-treated with shAR or with scrambled control (scr). After 72-h incubation, cells were treated with 20 μg/cm^2^ COM crystals for 24 h. **a** Quantitative real-time PCR (qRT-PCR) analysis of MΦs-associated cytokines in HK-2 and HKC-8 cells with/without knockdown of AR. **b** The level of CSF-1 in the CM of RTCs was detected by ELISA. **c** The level of CSF-1 in the RTCs lysates was detected by western blot. **d** qRT-PCR (left panels) and western blot (right panels) show CSF-1 knockdown efficiency in HK-2 and HKC-8 cells. **e** Knocking down AR in HK-2 and HKC-8 cells increased M0-MΦs migration, whereas knocking down CSF-1 interrupted the AR knockdown-mediated increase of M0-MΦs migration. **f** CM from AR-depleted RTCs led to increase mRNA levels of markers of M2 phenotypic MΦs (CD163 and CD206), whereas knocking down CSF-1 in RTCs interrupted AR knockdown-mediated M2-MΦs markers change. **g** Flow cytometry analysis showed that CM from AR-depleted RTCs led to increase expressions markers of M2 phenotypic MΦs (CD163 and CD206), whereas knocking down CSF-1 in RTCs interrupted AR knockdown-mediated M2-MΦs markers change. **h** Knocking down CSF-1 in HK-2 and HKC-8 partly reversed the AR knockdown-enhanced COM crystals phagocytic ability of MΦs. For **e, g** and **h**, quantifications are at the right. All quantitations are presented as mean ± SD. *P < 0.05, **P < 0.01, ***P < 0.001, ****P < 0.0001

To dissect the mechanisms of how targeting AR in renal epithelial cells can promote the MΦs recruitment and M2 polarization, we first screened commonly known cytokines related to MΦs recruitment and M2 polarization^15–18^. The results revealed that the expression of mRNA from some selective cytokines, including CSF-1, interleukin-10 (IL-10), and IL-34, were altered after manipulating the AR expression (Fig. 2a). Among these cytokines, we noticed that the CSF-1 mRNA expression was altered most significantly. Importantly, we also confirmed the CSF-1 protein expression of renal epithelial cells was also increased after knocking down AR (Fig. 2b, c), and when we replaced the THP-1 macrophages/HK-2 cells with RAW264.7 macrophages/M1 cells (Supplementary Fig 1. E-F). We therefore decided to further study the impact of CSF-1 on the AR-altered MΦs recruitment and M2 polarization.

Using an interruption approach, we confirmed that knocking down CSF-1 with shRNA could partially reverse the AR knockdown-increased M0-MΦs recruitment (Fig. 2d, e), and M2 polarization (Fig. 2f, g), suggesting that targeting AR in renal epithelial cells may function via increasing the CSF-1 expression in the CM to promote MΦs recruitment and M2 polarization. Importantly, the results from COM phagocytosis assay also supported that targeting AR in renal epithelial cells could enhance the MΦs COM crystals phagocytic ability and is CSF-1 dependent (Fig. 2h). Similar results were also obtained when we used the second CSF-1-shRNA (Supplementary Fig, 2E-F), as well as when we replaced the THP-1 macrophages/HK-2 cells with RAW264.7 macrophages/M1 cells (Supplementary Fig. 1G-I).

Together, results from Figs. 2a–h and Supplementary Fig. 1E-I and 2E-F suggest that targeting AR in renal epithelial cells may function via upregulating the CSF-1 signals to enhance the MΦs recruitment and M2 polarization.

### Mechanism dissection of how AR suppresses CSF-1 protein expression: via upregulating the miR-185-5p expression

**Fig. 3:**
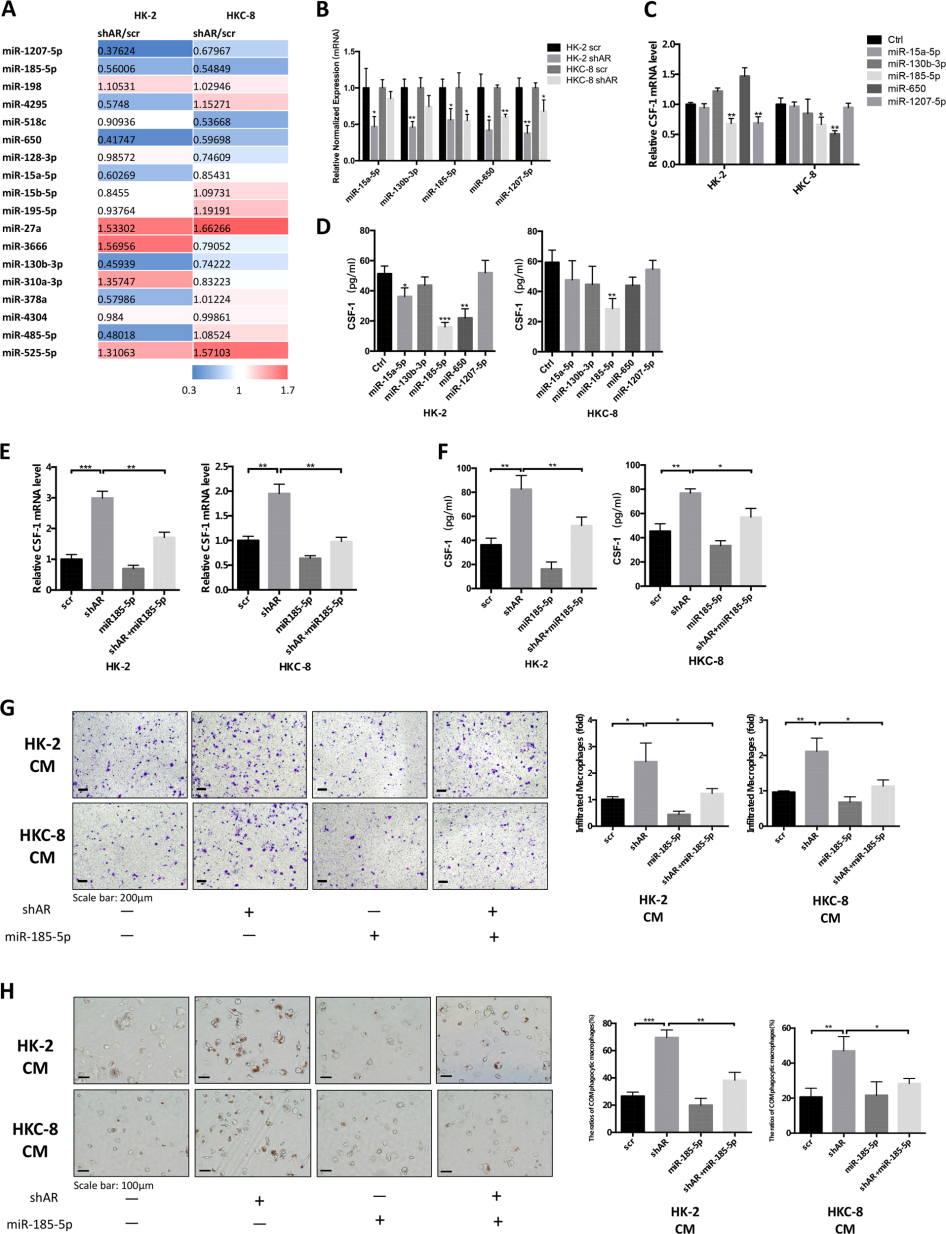
Androgen receptor (AR) modulates CSF-1 via upregulation of miR-185-5p in renal tubular epithelial cells (RTCs) exposed to calcium oxalate monohydrate (COM) crystals. **a** Eighteen potential miRNAs candidates were screened by quantitative real-time PCR (qRT-PCR) assay in HK-2 cells and HKC-8 cells based on their response to knocking down AR. Cells were pre-treated with 20 μg/cm^2^ COM crystals for 24 h. **b** The qRT-PCR analysis of five miRNAs in both HK-2 and HKC-8 cells with shAR compared with control. **c, d** HK-2 and HKC-8 cells were virally transduced with miR-15a-5p, miR-130b-3p, miR-185-5p, miR-650, and miR-1207-5p. Cells were then exposed to 20 μg/cm^2^ COM crystals for 24 h. Total RNAs (**c**) were analyzed for CSF-1 by qRT-PCR. The protein expression levels (**d**) of CSF-1 in the CM of HK-2 and HKC-8 cells were assessed by ELISA. **e** The mRNA levels of CSF-1 were determined by qRT-PCR analysis after co-transfection with shAR and miR-185-5p (vs. scramble). **f** The protein levels of CSF-1 in RTCs CM were determined by ELISA after co-transfection with shAR and miR-185-5p (vs. scramble). **g** M0-MΦs migration to the CM from HK-2 cells (upper) and HKC-8 cells (lower) with four groups (scramble, shAR, miR-185-5p, and shAR + miR-185-5p). **h** Overexpressing miR-185-5p in HK-2 cells and HKC-8 cells partly reversed the AR knockdown-enhanced COM crystals phagocytic ability of MΦs. For **g** and **h**, quantifications are at the right. All quantitations are presented as mean ± SD. *P < 0.05, **P < 0.01, ***P < 0.001

To dissect the detailed mechanism(s) how knocking down AR can increase the CSF-1 protein expression, we focused on miRNA regulation as we failed to identify a potential androgen response element (ARE) on the CSF-1 promoter region that could be validated by the chromatin immunoprecipitation (ChIP) in vivo binding assay (data not shown). We then selected 18 miRNAs that might be able to target CSF-1 via binding to the 3ʹ untranslated region (3ʹ-UTR) from analysis results of databases (Targetscan, miRanda, RNA22, and miRWalk) and applied quantitative real-time PCR (qRT-PCR) to determine whether any of these potential miRNA candidates might be the targets after knocking down AR in renal epithelial cells. The results revealed that, in cells incubated with 20 μg/cm^2^ COM crystals, the expressions of five miRNAs (miR-15a-5p, miR-130b-3p, miR-185-5p, miR-650, and miR-1207-5p) were significantly suppressed after knocking down AR in HK-2 and HKC-8 cells (Fig. 3a, b).

We further assayed the consequences on CSF-1 expression after adding these five miRNAs to the HK-2 and HKC-8 cells, and results suggest that miR-185-5p is the best candidate for further study since altering this miRNA significantly decreased CSF-1 expression (Fig. 3c, d).

As expected, results from the interruption approach via adding miR-185-5p led to partially reverse AR-shRNA-enhanced CSF-1 expression in HK-2 and HKC-8 cells (Fig. 3e, f). The consequences of such reversion may then lead to partially reverse the targeted AR-enhanced M0-MΦs recruitment and COM crystals phagocytic ability of MΦs (Fig. 3g, h). Similar results were also observed when we replaced the THP-1 macrophages/HK-2 cells with RAW264.7 macrophages/M1 cells (Supplementary Fig. 1J-N).

Together, results from Fig. 3a–h and Supplementary Fig. 1J-N, using multiple macrophages and renal epithelial cells, suggest AR can suppress CSF-1 protein expression via upregulating the miR-185-5p expression in the renal epithelial cells.

### Mechanism dissection of how AR increases miR-185-5p expression

**Fig. 4:**
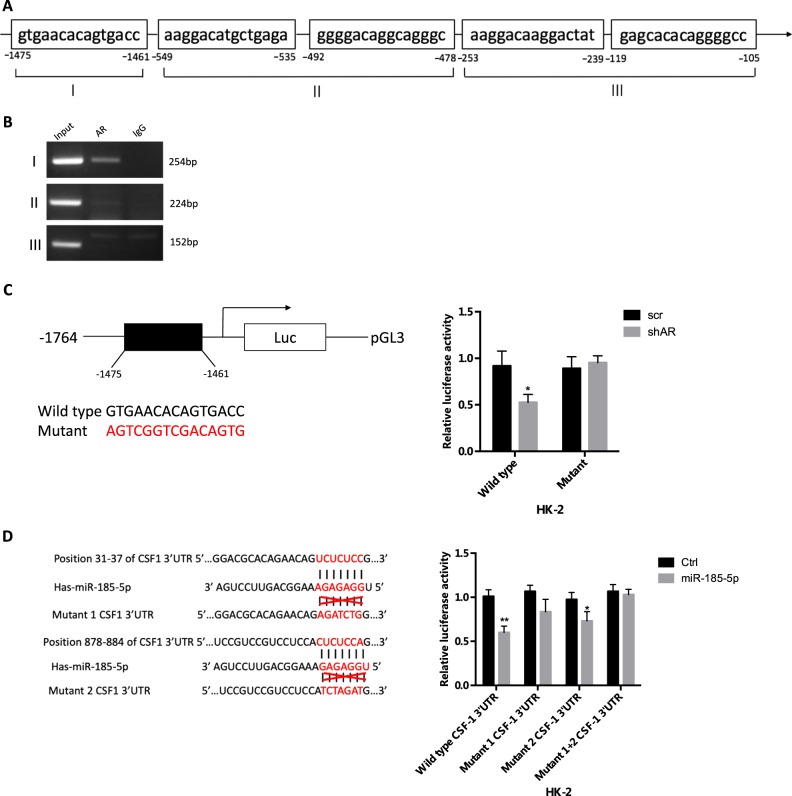
Mechanism dissection how androgen receptor (AR) regulates miR-185-5p expression and how miR-185-5p regulates CSF-1 expression. **a** Predicted localization of androgen response elements (AREs) in miR-185-5p promoter region. **b** Chromatin immunoprecipitation (ChIP) analysis of AR binding to miR-185-5p promoter in HK-2 cells pre-treated with 20 μg/cm^2^ COM for 24 h. **c** Luciferase activity of reporter constructs harboring wild type or mutated miR-185-5p promoter in HK-2 cells transfected with plasmids encoding shAR. The cells were pre-treated with 20 μg/cm^2^ COM for 24 h. **d** Co-transfection of CSF-1 3ʹ-UTR constructs containing wild type or mutant 1 or mutant 2 seed regions with miR-185-5p into HK-2 cells and luciferase assay was applied to detect the luciferase activity. All quantitations in **c** and **d** are presented as mean ± SD, *P < 0.05, **P < 0.01

To further dissect the molecular mechanism how AR can upregulate miR-185-5p expression in the renal epithelial cells exposed to COM crystals, we searched for potential AREs on the 2 kb 5ʹ promoter region of miR-185-5p using the JASPAR database. The results identified five putative AREs located within the miR-185-5p promoter region (Fig. 4a), and results from the ChIP assay revealed that AR could bind to the ARE located at 1461–1475-bp upstream of the transcriptional start site of miR-185-5p in HK-2 cells (Fig. 4b). Furthermore, using the luciferase assay to examine the 1.7 kb miR-185-5p promoter region that was linked to the pGL3-basic Vector (Promega), we found that knocking down AR with AR-shRNA could decrease the luciferase expression of the miR-185-5p wild-type (WT) promoter construct, but not the ARE mutant promoter construct, in HK-2 cells exposed to COM crystals (Fig. 4c).

Together, results from Fig. 4a–c suggest AR can upregulate miR-185-5p expression via transcriptional regulation with binding to ARE on its 5ʹ promoter in the renal epithelial cells.

### Mechanism dissection of how AR increased miRNA-185-5p led to suppressing the CSF-1 expression

To dissect the mechanism how AR increased miR-185-5p can suppress the CSF-1 expression, we searched for the potential binding sites of miR-185-5p located on the 3ʹ-UTR of CSF-1 mRNA, and identified two predicted miRNA-responsive-elements that matched the seed sequence of miR-185-5p in the 3ʹ-UTR of CSF-1 gene (Fig. 4d). We then inserted a 1705-bp fragment from the CSF-1 3ʹ-UTR with the predicted miR-185-5p target sites into a dual-luciferase reporter backbone psiCHECK^TM^-2 downstream of the Renilla luciferase open reading frame, as well as a mutated version at the predicted target sites (Fig. 4d, left). The luciferase assay results revealed that miR-185-5p could suppress luciferase expression of the WT CSF-1 3ʹ-UTR construct, the mutant 1 and mutant 2 CSF-1 3'-UTR constructs, but not the mutant 1+2 CSF-1 3ʹ-UTR construct, suggesting that miR-185-5p could directly target the CSF-1 3ʹ-UTR to decrease its expression (Fig. 4d, right).

### In vivo study using mouse model to determine the AR role in the development of CaOx crystals deposition

**Fig. 5:**
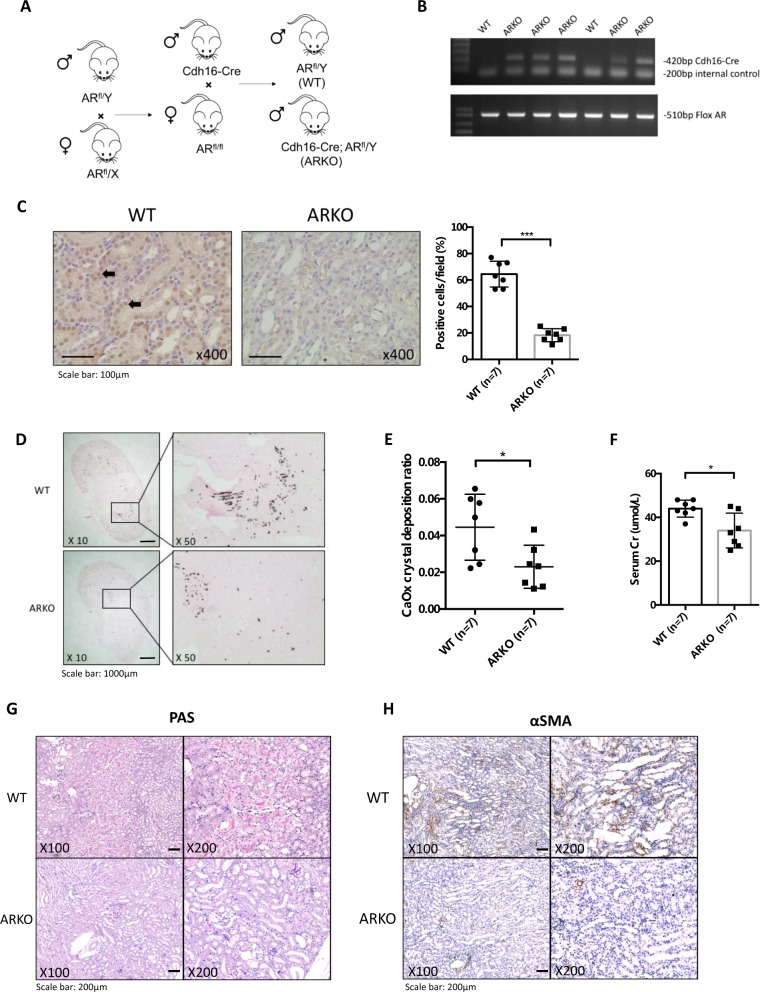
Loss of renal tubular epithelial androgen receptor (AR) gene decreased the intrarenal calcium oxalate (CaOx) crystals deposition in glyoxylate-induced CaOx deposition mouse model. **a** Renal tubular epithelial ARKO mouse breeding scheme. Male mice with Cre coding sequence under the control of the mouse cadherin 16 (Cdh16) promoter were bred with female flox AR mice (AR^fl/fl^) to generate wild type (WT, non-carrier for Cdh16-Cre) and Cdh16-Cre/ARKO mice (Cdh16-ARKO). **b** Tail genomic DNA was isolated for genotyping by PCR using primers flanking AR exon 2 and Cre primers. **c** AR protein expression (left panels) was detected in male kidneys of WT and Cdh16-ARKO mice by immunohistochemistry (IHC). Black arrows indicate cells expressing AR protein. Quantifications of AR-positive cells in the mouse renal tissues were shown at right. **d** Glyoxylate-induced intrarenal CaOx crystals deposition was compared between WT and Cdh16-ARKO male mice. **e** Quantitation (mean ± SD) of intrarenal CaOx crystals deposition in each kidney section. Higher numbers of intrarenal CaOx crystals were found in the WT mice than in the Cdh16-ARKO mice. **f** Serum creatinine (Cr) level was compared between WT and Cdh16-ARKO male mice after glyoxylate treatment. **g** Representative histologic images of periodic acid–Schiff (PAS)-stained kidney sections from WT and ARKO mice. **h** Immunostaining of α-smooth muscle actin (α-SMA) in kidney sections from WT and ARKO mice. *P < 0.05, ***P < 0.001 compared with WT mice

To confirm the above in vitro cell line results with the in vivo mouse model, we generated renal tubule-specific AR knockout mice (Cdh16-ARKO) by breeding floxed AR mice with Cdh16 promoter-driven Cre transgenic mice, in which Cre expression is limited to the renal tubules, including Bowman’s capsule, proximal tubules, and loop of Henlé and distal tubules^19^ (Fig. 5a). Genotyping results show that Cdh16-ARKO mice had both Cre and floxed AR alleles (Fig. 5b). To verify the loss of AR in renal tubules of Cdh16-ARKO mice at the protein level, we performed immunohistochemical (IHC) analysis and demonstrated that the AR-positive renal epithelial cells were drastically reduced in Cdh16-ARKO mice in comparison with WT mice (Fig. 5c).

We then utilized i.p. injections of glyoxylate to induce renal CaOx crystals deposition to investigate the renal tubular AR effect on the CaOx crystals deposition. Male mice were given daily i.p. injections of glyoxylate (80 mg/kg/day). Six days after glyoxylate injections, the mice were euthanized to determine the CaOx crystals deposition. As shown in Fig. 5d, e, Cdh16-ARKO mice had fewer CaOx crystals deposition than the WT mice.

We next examined the effect of targeting AR on renal function and histologic changes in the mice after inducing the CaOx crystals deposition. The results revealed that Cdh16-ARKO mice have a decrease in serum creatinine as compared with the WT mice (Fig. 5f). In addition, deleterious tissue changes in the medulla (tubular necrosis and casts formation) by periodic acid-Schiff (PAS) staining were staining were more evident in kidneys from WT mice than in kidneys from Cdh16-ARKO mice (Fig. 5g).

We further evaluated renal fibrosis by IHC staining of αSMA, and results indicated that kidneys from Cdh16-ARKO mice exhibited a decreased number of myofibroblasts, compared with kidneys from WT mice (Fig. 5h).

Together, these results from Fig. 5a–h suggest that targeting the renal epithelial AR may lead to reduction of glyoxylate-induced renal CaOx crystals deposition.

### Increased CSF-1 expression and M2-polarized MΦs in the Cdh16-ARKO mouse with glyoxylate-induced CaOx crystals deposition

**Fig. 6:**
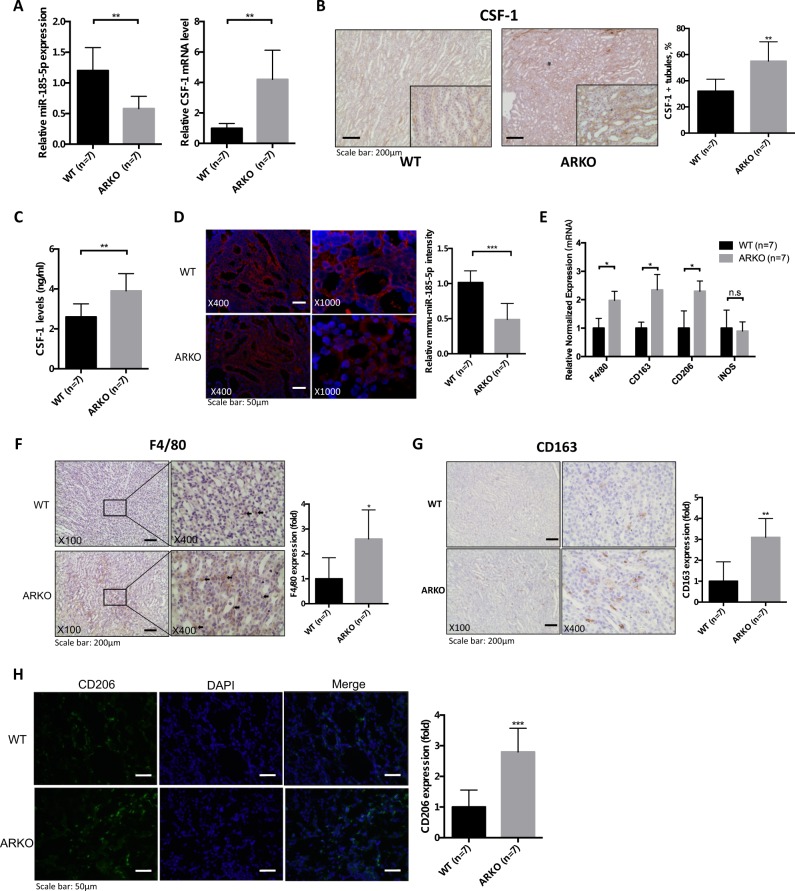
Loss of androgen receptor (AR) increased renal CSF-1 expression and M2 macrophage infiltration in glyoxylate-induced intrarenal calcium oxalate (CaOx) crystals deposition mouse model. **a** The quantitative real-time PCR (qRT-PCR) analysis of CSF-1 gene and miR-185-5p expression in mice kidneys. **b** (IHC staining of mouse kidney tissues showing that loss of AR in Cdh16-ARKO mice increased the CSF-1 expression. Quantification of CSF-1 positive renal tubules is shown in the right panel. **c** Kidney CSF-1 levels in WT and Cdh16-ARKO mice. CSF-1 levels in kidney homogenates determined by ELISA. **d** Representative MISH staining results for mmu-miR-185-5p in kidney tissues from WT or Cdh16-ARKO mice. **e** AR deletion in the renal tubule led to increased mRNA levels of markers of pan-MΦs and M2-like MΦs, including F4/80, CD163, CD206 (mannose receptor), but not INOS, in kidneys from mice with glyoxylate injection for 6 days. **f** Immunostaining indicated increased renal F4/80-positive MΦs in the renal corticomedullary junction from Cdh16-ARKO mice at 6 days after glyoxylate injection. **g** Immunostaining indicated increased renal CD163-positive MΦs in the renal corticomedullary junction from Cdh16-ARKO mice at 6 days after glyoxylate injection. **h** Immunofluorescence indicated increased renal CD206-positive MΦs in the Cdh16-ARKO mice at 6 days after glyoxylate injection. For B, D, F, G and H, quantitations are at the right and all quantitations are mean ± SD, *P < 0.05, **P < 0.01, ***P < 0.001, n.s. not significant compared with WT mice group

We then assayed the CSF-1 mRNA and miR-185-5p expression in kidney tissues via qRT-PCR assay, and the results showed that a higher renal CSF-1 mRNA and a lower miR-185-5p expression were detected in Cdh16-ARKO mice compared with WT mice after glyoxylate treatment (Fig. 6a). Importantly, results from IHC, enzyme-linked immunosorbent assay (ELISA), and microRNA in situ hybridization (MISH) assay also showed higher CSF-1 protein expression (Figs. 6b, c) and lower miR-185-5p expression (Fig. 6d) in Cdh16-ARKO mice.

We next assayed the recruitment of macrophages in renal tissues, and found Cdh16-ARKO mice had higher renal expression of F4/80 (a widely used marker of murine macrophages^20^) vs. WT mice at the mRNA level (Fig. 6e) and at the protein level (Fig. 6f).

We then examined the population of M1-MΦs vs. M2-MΦs in the renal tissues, and showed that the renal mRNA expressions of M2-MΦs markers, including the CD163 and CD206, were significantly higher in Cdh16-ARKO mice as compared with those found in WT mice (Fig. 6e). In contrast, the renal mRNA expression of iNOS, a M1-MΦs marker, was not significantly different between Cdh16-ARKO and WT mice (Fig. 6e). Importantly, IHC and immunofluorescence staining further confirmed these differences at the protein level (CD163 in Fig. 6g and CD206 in Fig. 6h) in Cdh16-ARKO vs WT mice.

Together, results from Fig. 6a-h suggest that Cdh16-ARKO mice have reduced CaOx crystals deposition with increased M2 macrophage recruitment.

### In vivo study using ASC-J9® to degrade AR in the hydroxy-l-proline (HLP)-induced CaOx crystals deposition rat model led to the reduction of CaOx crystals deposition

All in vivo studies using the ARKO mouse model (Figs. 5, 6) demonstrated that AR induced CaOx crystals deposition via altering the renal M2 macrophages recruitment/polarization. This mouse model demonstrated how AR may impact the development of intrarenal CaOx crystals deposition in vivo^21^. To substantiate these results in another animal model, we used rats fed with HLP for 8 weeks to induce intrarenal CaOx crystals deposition^22^, as recent studies indicated this rat fed with HLP more closely modeled human intrarenal CaOx crystals deposition without inducing the acute kidney injury^21,22^.

**Fig. 7:**
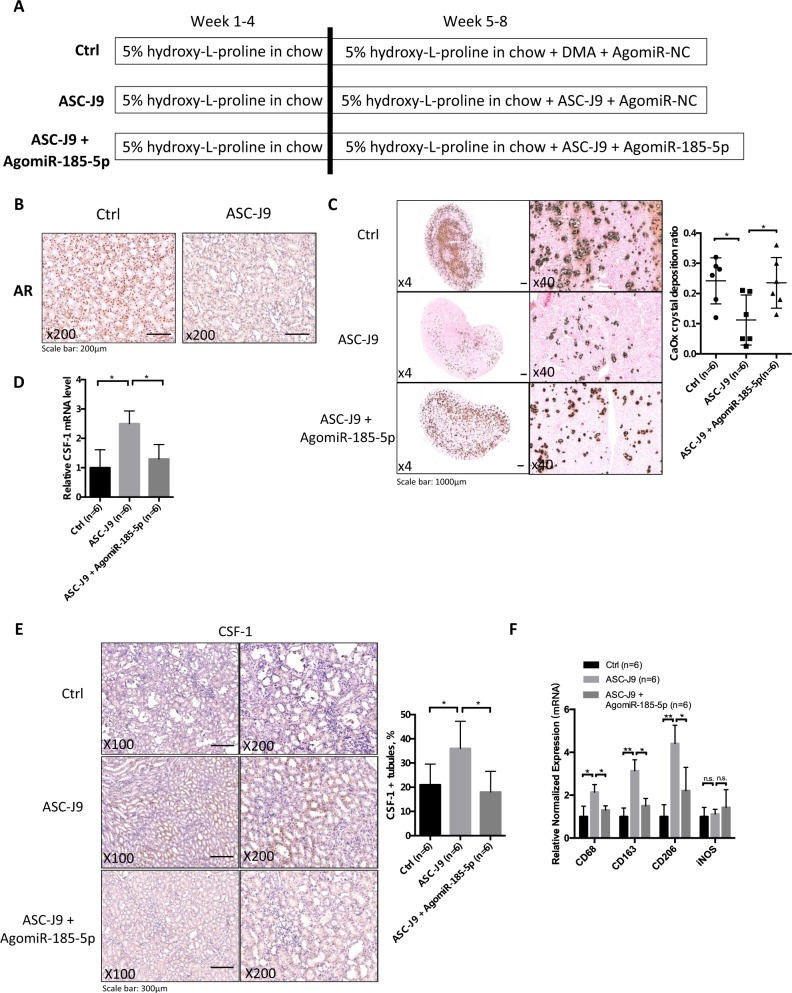

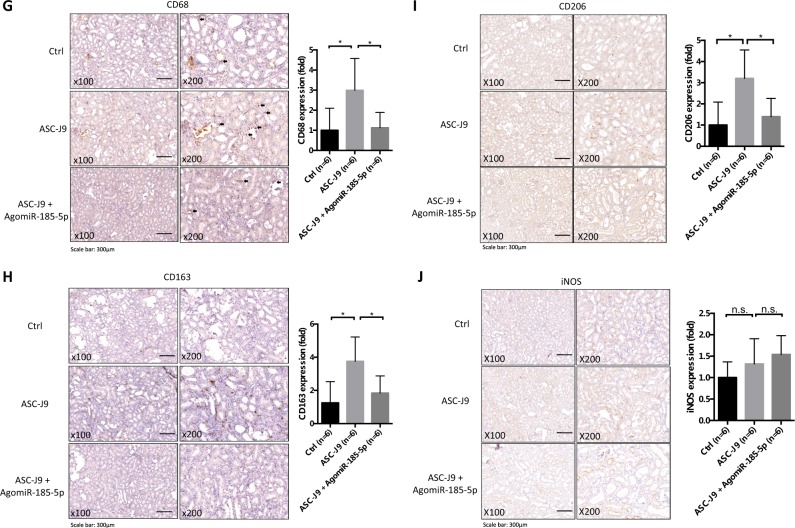
Targeting androgen receptor (AR) with ASC-J9 promotes intrarenal calcium oxalate (CaOx) crystals elimination in rat model via increasing renal CSF-1 expression and M2 macrophages infiltration. **a** A diagram describing the injection schedule for ASC-J9® (ASC-J9) and AgomiR-185-5p. **b** IHC showing AR degradation in kidney tissue of rat. Rats were injected i.p.with ASC-J9 (37.5 mg/kg) every other day for 4 weeks. Control rats were injected with vehicle, N,N-dimethylacetamide. **c** Crystals staining showing the deposition area of CaOx crystals in kidney tissues of the rats treated with/without ASC-J9 and AgomiR-185-5p. **d** Targeting AR by ASC-J9 increased the renal mRNA level of CSF-1, and adding miR-185-5p partially reversed the effect of ASC-J9-enhanced CSF-1 expression. **e** Immunohistochemical (IHC) staining of kidney tissues showing that targeting AR by ASC-J9 in rats increased the CSF-1 expression, and adding miR-185-5p reversed the effect of ASC-J9. **f** AR deletion by treatment of ASC-J9 led to increased mRNA levels of markers of pan-MΦs (CD68) and M2-like MΦs (CD206 and CD163) in kidney from rats fed with HLP for 8 weeks. Adding miR-185-5p led to partially reverse the effect of ASC-J9. **g** Immunostaining indicated increased renal CD68-positive MΦs in the renal corticomedullary junction from ASC-J9-treated rats fed with HLP for 8 weeks. **h** Immunostaining indicated increased renal CD163-positive MΦs in the renal corticomedullary junction from ASC-J9-treated rats fed with HLP for 8 weeks. Adding miR-185-5p led to partially reverse the effect of ASC-J9. **i** Immunostaining indicated increased renal CD206-positive MΦs from ASC-J9-treated rats fed with HLP for 8 weeks. Adding miR-185-5p led to partially reverse the effect of ASC-J9. **j** Immunostaining indicated renal iNOS expression was comparable between ASC-J9 treated and control rats fed with HLP for 8 weeks. For c, e, g-j, quantitations are at the right and all quantitations are mean ± SD. One-way ANOVA followed by Bonferroni multiple comparison test was used. *P < 0.05, **P < 0.01, n.s. not significant

We also replaced the knockout AR strategy by treating with the small molecule, ASC-J9®, which can selectively degrade AR^23–28^ in this second animal model. We first fed rats with chow mixed with 5% HLP (weight/weight, HLP/chow) for 8 weeks. After 4 weeks’ administration of HLP, rats were i.p. injected with ASC-J9® (37.5 mg/kg/48 h), or control vehicle (N,N-dimethylacetamide, DMA), and AgomiR-185-5p or AgomiR-negative control (NC) (15 nmol/kg/week) for 4 weeks (see details in Fig. 7a). The results revealed that ASC-J9® could degrade AR in kidney as compared with control mice that received vehicle (Fig. 7b). Importantly, using Pizzolato staining to examine the CaOx crystals deposition in rat kidney, we found fewer CaOx crystals deposition in the ASC-J9®-treated rats than those in the vehicle control-treated rats.

As expected, results from an interruption approach via adding miR-185-5p can then partially reverse/block the ASC-J9®-reduced CaOx crystals deposition (Fig. 7c).

Results from qRT-PCR assay also revealed a higher renal CSF-1 mRNA expression in the ASC-J9®-treated rats compared with control rats (Fig. 7d), and IHC staining also showed higher CSF-1 protein expressions in the ASC-J9® treated rats (Fig. 7e). Adding miR-185-5p led to reverse the effect of ASC-J9-enhanced CSF-1 expression.

We next assayed the recruitment of macrophages in renal tissues, and found ASC-J9® treated rats had higher renal expression of CD68 (a widely used marker of rat macrophages^20^) vs. control kidneys at the mRNA level (Fig. 7f) and at the protein level (Fig. 7g), and adding miR-185-5p could then lead to partially reverse/block the ASC-J9®-enhanced recruitment of macrophages.

Finally, we examined the expression of M1-MΦs and M2-MΦs in the renal tissues, and results revealed that the renal mRNA expression of M2-MΦs markers was significantly higher in ASC-J9® treated rats as compared with those found in control rats (Fig. 7f). In contrast, we found little difference in the renal mRNA expression of iNOS, a M1-MΦs marker, between the two groups (Fig. 7f). Importantly, IHC also confirmed these differences at the protein level (CD163 in Fig. 7h and CD206 in Fig. 7i as well as iNOS in Fig. 7j) in ASC-J9® treated rats vs. control rats. As expected, adding miR-185-5p could then lead to partially reverse/block the ASC-J9® induced M2 macrophages polarization.

Taken together, the results from in vivo study using rat model (Fig. 7a–j) demonstrate that targeting AR with ASC-J9® is an effective new therapeutic approach to better suppress the CaOx crystals deposition via altering the AR-mediated miR-185-5p/CSF-1/M2 macrophages recruitment and polarization signaling.

## Discussion

**Fig. 8:**
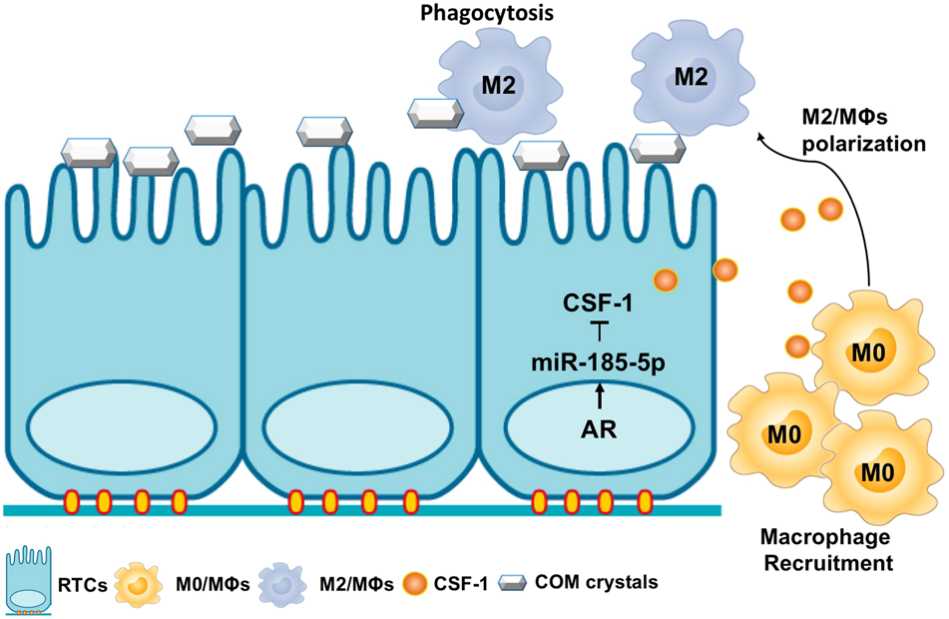
Scheme. The scheme diagram summarizes the pathway described: androgen receptor (AR) signaling could suppress CSF-1 expression via upregulation of miR-185-5p in renal tubular epithelial cells (RTCs) in response to calcium oxalate monohydrate (COM) crystals stimuli. Tissue production of CSF-1 can induce M0-MΦs recruitment from the blood, and skew MΦs toward an M2 phenotype, which has a high crystals phagocytic ability

The kidney is susceptible to crystals/stone formation as urine ion concentrations favor supersaturation, which promotes crystalization and crystals formation/deposition that may lead to acute kidney injury, nephrocalcinosis and chronic kidney disease^29^. In the present study, we found AR could alter the CaOx crystals deposition via suppressing the macrophages recruitment and polarization of macrophages towards the M2 phenotype (Fig. 8). These findings may provide valuable information for future potential approaches via altering the AR-meditated macrophages recruitment/polarization signals to suppress the CaOx crystals deposition. These results are in agreement with previous studies indicating that infiltrating M2-MΦs have an important physiological role in decreasing the CaOx crystals deposition via engulfing the CaOx crystals fragments^14^.

The renal CaOx crystals deposition may start with the formation of the CaP nuclei in the renal interstitium that is gradually developed into the Randall plaques, and erosion of these plaques into the urinary space may then promote the heterogeneous nucleation with supersaturated CaOx to form the kidney stones^30,31^. In contrast, some kidney stone formers have their crystals initially arise within the the tubule lumens of terminal collecting ducts. Since all current available mouse and rat models for the kidney stone diseases developed few Randall plaques, their kidney crystals formation may be more close to those kidney stone patients with hyperoxaluria.

It is now well recognized that macrophages serve numerous functions and manifest a variety of phenotypes^32^. Taguchi et al.^5^ reported that M2 macrophages might suppress CaOx crystals deposition in M2-deficient mice through phagocytosis of the renal CaOx crystals with inhibiting inflammation. In contrast, M1 macrophages are not able to remove intrarenal CaOx deposits and might further accelerate oxidative-induced kidney injury via promoting the renal inflammation. Here we showed that exposure of macrophages to the CM of renal tubular cells with AR-shRNAs could lead to increased expression of M2 markers and alter their crystals phagocytic ability in vitro. Importantly, results from two animal models also showed that targeting AR in the renal cells could decrease CaOx crystals deposition with increasing the M2 macrophages infiltration in the kidneys.

CSF-1 plays key roles in renal macrophages proliferation and polarization^33^, and is well known for its role in the bone marrow to facilitate the production of blood monocytes, the precursors for the tissue macrophages^34,35^. Renal tubular cells were identified as the major site of CSF-1 production^34,36^. Wang et al.^36^ established proximal tubule production of CSF-1 as important for polarization of renal MΦs and recovery from acute kidney injury. CSF-1 has also been identified as a powerful regulator for macrophages proliferation, differentiation, and polarization for suppression of CaOx crystals deposition^5,6,14,37,38^. Sergei et al.^6^ demonstrated in vitro that MΦs differentiated with CSF-1 were able to effectively phagocytize renal CaOx crystals <200 μm. Taguchi et al.^5^ reported that a lack of CSF-1 production in the op/op mouse leads to a markedly increased intrarenal CaOx crystals deposition and substantially reduced renal M2-like macrophage populations, and adding CSF-1 could then reverse such deficiency.

Here, we first confirmed that CSF-1 derived from renal tubular cells can play a key role in altering the renal macrophages and CaOx crystals deposition. We further proved that AR could modulate CSF-1 expression in renal epithelial cells, suggesting that it is possible to alter the CSF-1 expression via targeting AR to suppress the intrarenal CaOx crystals deposition.

ASC-J9® is a newly developed AR degradation enhancer that can selectively degrade AR in some cells. Recent studies have demonstrated that ASC-J9® can suppress many AR-mediated diseases, including prostate cancer, liver cancer, and spinal and bulbar muscular atrophy neuron disease^25–28,39^. Our in vivo results showing ASC-J9® can also suppress CaOx crystals deposition via altering the M2 macrophages recruitment/polarization may provide a novel approach to suppress CaOx crystals deposition.

In summary, our study demonstrated that that renal tubular AR could function through altering the macrophage recruitment and M2 polarization to influence the CaOx crystals deposition via altering the miRNA-185-5p/CSF-1 signaling, which may provide clinicians a novel therapy to better suppress the renal CaOx crystals deposition.

## Materials and methods

### Cell lines

The human proximal tubular epithelial HK-2 cells, human embryonic kidney 293 cells (HEK-293), human monocyte THP-1 cells, mouse macrophage RAW264.7 cells and mouse cortical collecting duct M1 cells were purchased from the American Type Culture Collection (ATCC) (Rockville, MD). The human proximal tubular epithelial HKC-8 cells were kindly provided by Dr. Syed Khundmiri of the University of Louisville (Louisville, Kentucky). The HK-2, HKC-8, HEC-293, RAW-264.7, and M1 cells were maintained in Dulbecco’s modified Eagle’s media with 10% fetal bovine serum (FBS) and 1% penicillin/streptomycin. The THP-1 cells were cultured in RPMI-1640 media supplemented with 10% FBS. The THP-1 cells were differentiated to macrophages by treating with 100 ng/ml PMA for 3 days before being used in experiments.

### Lentiviral expression plasmid construction and virus production

The shAR (pLKO.1-puro-shAR) was constructed with target sequence 5ʹ-GTCGCGACTACTACAACTT-3ʹ and shCSF-1 (pLKO.1-puro-shCSF-1) was constructed with target sequence 5ʹ-TCTCCTGGTACAAGACATAAT-3ʹ according to Addgene’s protocol. The pLKO.1-shAR, pLKO.1-shCSF-1, pLVTHM-miR-15a-5p, pLVTHM-miR-130b-3p, pLVTHM-miR-185-5p, pLVTHM-miR-650, or pLVTHM-miR-1207-5p, the psAX2 packaging plasmid, and pMD2G envelope plasmid, were then transfected into HEK-293 cells using the standard calcium chloride transfection method for 48 h to get the lentivirus soup. The concentrated lentivirus soup was used immediately or frozen at −80 ℃ for later use.

### COM crystals preparation and staining

COM crystals were prepared as described previously^40^. Briefly, 0.5 mM Na_2_C_2_O_4_ was mixed with 5 mM CaCl_2_ in a buffer containing 90 mM Tris-HCL/pH7.4 and 10 mM NaCl. For staining, COM crystals were crystallized in the presence of 22.5 μg/mL Ponceau-S (Sigma-Aldrich; St. Louis, MO). The mixture was incubated at room temperature overnight and the COM crystals were harvested by a centrifugation at 2000 rpm for 5 min. The crystals pellets were washed in absolute methanol and then collected by another centrifugation at 2000 rpm for 5 min. The size of crystals generated by this protocol was 5–15 μm. The crystals were then dried and decontaminated by UV light radiation overnight.

### Renal tubular cells exposure to COM crystals and collection of the CM

HK-2, HKC-8, and M1 cells (shAR or scr, or treated with varying concentrations of DHT or vehicle (ethanol)) were placed in 10 cm culture dishes, grown until close to confluent, washed with phosphate-buffered saline (PBS), and incubated with 20 μg/cm^2^ COM crystals. After 24 h of incubation, the cells were collected for qRT-PCR or western blot experiments, and the CM were collected for further experiments.

### Macrohahage recruitment assay

Chambers with 5.0-μm polycarbonate filter inserted in 24-well plates were used in the quantitative cell migration assays. In all, 1 × 10^5^ PMA-differentiated-THP-1 macrophages (M0-MΦs) or mouse RAW264.7 macrophages were plated onto the upper chambers, and the lower chambers were filled with the RTCs’ CM. After 18- to 20-h incubation, the non-migrated cells in the upper chamber were removed and cells migrated into the membrane were fixed with methanol, stained with crystal violet, and photographed under an inverted microscope. Cell numbers were counted in five randomly chosen microscopic fields per membrane.

### Macrophage co-culture with the RTCs CM and evaluation of crystals phagocytosis

The CM from RTCs were mixed with fresh RPMI media at a ratio of 1:1 and then added to M0-MΦs or RAW264.7 macrophages for 3 days. To determine gene (MΦs’ markers) expression, MΦs were collected in Trizol and analyzed as described below. For evaluation of COM crystals phagocytosis, the cells were maintained in normal RPMI media overnight and then treated with 15 μg/cm^2^ Ponceau-S labeled COM crystals for 24 h. The percent of macrophages containing internalized Ponceau-S labeled COM crystals was then analyzed by optical microscopy.

### Quantification of CSF-1 in the CM

CM was collected from HK-2, HKC-8, and M1 cells. ELISA kit (BOSTER) was used to measure CSF-1 concentrations in the CM according to the manufacturer’s instructions.

### Quantification of CSF-1 in mice kidney homogenates

We evaluated the levels of CSF-1 in kidney homogenates using an ELISA method as previously detailed^41^. In brief, the kidney tissues were homogenized by using a MixMill 300 (Qiagen). The protein (200 μg/tissue) of each homogenate was determined using the BCA protein Asaay Kit. (Pierce). ELISA kit (BOSTER) was used to measure CSF-1 concentrations according to the manufacturer’s instructions.

### RNA extraction and qRT-PCR analysis

Total RNA was extracted by Trizol reagent (Invitrogen, CA) according to the manufacturer’s instructions. RNAs (1 μg) were subjected to reverse transcription using Superscript III transcriptase (Invitrogen). The qRT-PCR was conducted using a Bio-Rad CFX96 system with SYBR green to determine the mRNA expression level of a gene of interest. Expression levels were normalized to the expression of GAPDH, U6, or RPL32 RNA. Primers used are in the Supplementary Table 1.

### Antibodies

The antibodies used in this study are listed in Supplementary Table 2.

### Flow cytometry analysis

The CM from RTCs were mixed with fresh RPMI media at a ratio of 1:1 and then added to M0-MΦs or RAW264.7 macrophages for 3 days. The identification and characterization of macrophage polarization were performed using flow cytometry. Cells were harvested, pre-incubated with the Fc receptor binding inhibitor antibody (Bioscience™) for 10 min and thereafter incubated with phycoerythrin (PE)-conjugated mouse anti-human CD206 and Fluorescein (FITC)-conjugated mouse anti-human CD163 for 60 min. After washing the cells with flow cytometry staining buffer (Bioscience™), the expression of the cell surface markers CD206 and CD163 was analyzed using flow cytometry on FACSCalibur™ (BD Biosciences, USA).

### Western blot assay

Total protein was extracted by RIPA buffer containing 1% protease inhibitors (Amresco, Cochran, USA). The immuno-positive bands were visualized with an ECL chemiluminescent detection system (Thermo Scientific), and the images were transferred to the Bio-Rad imaging system.

### ChIP assay

Cell lysates were pre-cleared sequentially with normal rabbit IgG (sc-2027, Santa Cruz) and protein A-agarose. Then, 2.0 μg anti-AR antibody was added to cell lysates and incubated at 4 ℃ overnight. For the negative control (NC), IgG was used. Specific primer sets designed to amplify a target sequence with the miRNA-185-5p promoter are listed in the Supplementary Table 3; PCR products were identified by agarose gel electrophoresis.

### Luciferase assay

The fragment of CSF-1 3ʹ-UTR containing two miR-185-5p binding sites (sites 1 and 2) was amplified by PCR. The forward primer was 5ʹ-CAGTAATTCTAGGCGATCGCTGGACGCACAGAACAGTC-3ʹ, and the reverse primer was 5ʹ-GATATTTTATTGCGGCCAGCTGTCGGCATCAGGACAGG-3ʹ. The 3ʹ-UTR of CSF-1 was constructed into psiCheck2 (Promega, Madison, WI, USA) by the Gibson assembly method. Cells were plated in 24-well plates and the complementary DNAs (cDNAs) were transfected using Lipofectamine (Invitrogen) according to the manufacturer’s instructions. Luciferase activity was measured by Dual-Luciferase Assay (Promega) according to the manufacturer’s instructions.

The miR-185-5p promoter region (1764 bp) was amplified from human genomic DNA by Phusion® High-Fidelity DNA Polymerase (NEB, Beverly, NY) and ligased into Pgl3-basic vector (Promega) by the Gibson assembly method. For the ARE mutation, we designed the primer as forward: 5ʹ-GACAGTGACCATGGCCAGGAGGAAG-3ʹ, reverse: 5ʹ-GACCGACTATGTGGTCCTACTAGAA-3ʹ; then used WT miR-185-5p promoter pGL3 plasmid as the template to run PCR and performed self-ligation. For luciferase assay, cells were plated in 24-well plates and the cDNA transfected using Lipofectamine3000 (Invitrogen) according to the manufacturer’s instructions. pRL-TK was used as internal control. Luciferase activity was measured by Dual-Luciferase Assay (Promega) according to the manufacturer’s manual.

### Generation of Cdh16-ARKO mice with renal CaOx crystals formation/adhesion

All of the mouse experiments were performed under protocols approved by the Institutional Animal Care and Use Committee of the University of Rochester Medical Center. We generated ARKO mice that lacked AR gene in the RTCs via mating loxP site-AR female transgene (AR^flox/flox^ C57/B6) mice with cadherin 16 promoter-driven Cre (Cdh16-cre; C57/B6; The Jackson Laboratory) male mice expressing heterozygous Cre recombinase gene under the control of a tissue-specific promoter, Ksp-Cdh16, which was exclusively expressed in RTCs^42^. Tail genotyping followed methods in previous reports^19,43^.

We then established the renal CaOx crystals formation/adhesion mouse model following the reported protocol^44^. The 8-week-old male mice were given daily intra-abdominal injections of 80 mg/kg glyoxylate for 6 days. All animals had free access to chow and water.

### Development of hyperoxaluria-induced intrarenal CaOx crystals deposition in rat and ASC-J9® treatment

Eight-week-old Sprague–Dawley male rats (180–200 g) were divided into three groups (six rats/group) and all were given chow mixed with 5% HLP (weight/weight HLP/chow) for 8 weeks. After 4 weeks administration of HLP, rats were injected with ASC-J9® (37.5 mg/kg/48 h i.p.) or control vehicle DMA, and AgomiR-185-5p or AgomiR-negative control (15 nmol/kg/week) for 4 weeks. The AgomiRs were synthesized by Ribobio Inc. (Guangzhou, China). Next, the rats were sacrificed, and kidneys were obtained for analyses.

### Observation of intrarenal CaOx crystals deposition

The renal crystals formation/adhesion was examined using Pizzolato staining, as described previously^45^, and quantitatively assessed using Image J software.

### Histology and IHC

Kidneys from mice or rats were fixed for 72 h with 10% buffered formalin before embedding in paraffin. Serial sections at 5 μm thick were obtained for histologic anal-ysis. Hematoxylin and eosin (H&E) staining involved standard procedures.

For IHC, sections were incubated with the primary antibodies. To enhance antigen exposure, the slides were treated with 10 mM sodium citrate/pH 6.0 at 98 °C for 20 min for antigen retrieval. The slides were incubated with endogenous peroxidase blocking solution, and then were incubated with the primary antibody at 4 ℃ overnight. After rinsing with PBS, the slides were incubated for 45 min with biotin-conjugated secondary antibody, washed, and then incubated with enzyme conjugate horseradish peroxidase (HRP)–streptavidin. Freshly prepared and 3,3'-diaminobenzidine (DAB) (Zymed, South San Francisco, CA) was used as substrate to detect HRP. Finally, slides were counterstained with H&E and mounted with aqueous mounting media. Positive cells were calculated as the number of immunopositive cells × 100% divided by total number of cells/field in 10 random fields at ×400 magnification.

The macrophages subtypes were detected by immunofluorescence staining for CD206 using frozen sections. Frozen sections (8 μm) were fixed in 10% buffered formalin for 15 min. After washing with PBS containing 0.3% Triton X-100 for 15 min, the sections were incubated with the primary antibody at 4 ℃ overnight. After rinsing with PBS, the sections were then incubated for 1 h at room temperature with Alexa Fluor-conjugated 488-secondary antibodies, 1∶1000 (ThermoFisher). Finally, the sections were washed, counterstained with nuclear marker 4',6-diamidino-2-phenylindole (DAPI) and wet mounted. All images were obtained using a fluorescence microscope.

### MISH assay

The in situ hybridization for miRNAs was performed on fixed paraffin-embedded sections as previously described^46,47^. Oligonucleotide probes complementary to mouse-miR-185-5p were purchased from the Exonbio Lab (Guangzhou, China). The probe sequences were as follows: 5ʹ-TCAGGAACTGCCTTTCTCTCCA-3ʹ. These oligonucleotides contain 2ʹ-fluoro-modified RNA residues (2ʹ-F RNA) at the 3, 6, 15, and 20 bases. Both 5ʹ and 3ʹ ends were labeled by digoxin (DIG). In general, 10 μm thick sections from kidney tissues were deparaffinized, dehydrated, and subsequently immersed in 0.2 M HCl for 15 min. Slides were then immersed in PBS solution. Proteinase K (working solution: 200 μg/ml in PBS) digestion was used to treat tissues at 37 ℃ for 5 min. After digestion, slides were immersed in RNase-free water for 5 min and air dried. The slides were then prehybridized in hybridization buffer (Exonbio Lab, Guangzhou, China) at 37 ℃ for 2 h, followed by the hybridization with probe at 37 ℃ for 48 h. After hybridization, slides were washed 2×SCC with 0.5% Tween-20 twice for 5 min at room temperature. DNA was counterstained with DAPI (1 mg/ml). Images of miRNA signals in slides were captured by fluorescent microscope. For quantitative assessments, the images were analyzed by using Image J software.

### Statistics

All statistical analyses were carried out with SPSS 13.0 (SPSS Inc., Chicago, IL). The data values were presented as the mean ± SD. The data were first analyzed for normal distribution by using Kolmogorov–Smirnov test. Differences in normally distributed variables were analyzed by two-tailed Student’s *t*-test or one-way analysis of variance (ANOVA) followed by Bonferroni multiple comparison test. Differences were assessed by Mann–Whitney *U*-test for non-normally distributed variables. A two-sided *P* < 0.05 was considered statistically significant.

## Supplementary information

Supplementary Tables

Supplementary figure legends

Supplementary Fig 1-1

Supplementary Fig 1 continues

Supplemental Figure 2
