# Supplementary material for: Loss of the androgen receptor suppresses intrarenal calcium oxalate crystals deposition via altering macrophage recruitment/M2 polarization with change of the miR-185-5p/CSF-1 signals
Source: Cell Death Dis. 2019 Mar 20;10(4):275. doi: 10.1038/s41419-019-1358-y (PMC6427030; doi:10.1038/s41419-019-1358-y)
Supplement: Supplementary file 1 — Supplementary Tables [file 41419_2019_1358_MOESM1_ESM.docx]

**Supplementary Table 1. Primers sequences used in PCR experiments**

| **Gene name** | **Forward primer (5` to 3`)** | **Reverse primer (5` to 3`)** |
| --- | --- | --- |
| AR (human) | CCAGGGACCATGTTTTGCC | CGAAGACGACAAGATGGACAA |
| CD163 (human) | TTTGTCAACTTGAGTCCCTTCAC | TCCCGCTACACTTGTTTTCAC |
| CD163 (mouse) | TGTGCAGTAACGGCTGGAG | ATCATGTTTGCAGTCCCAAAGA |
| CD206 (human) | GGGTTGCTATCACTCTCTATGC | TTTCTTGTCTGTTGCCGTAGTT |
| CD206 (mouse) | CTCTGTTCAGCTATTGGACGC | TGGCACTCCCAAACATAATTTGA |
| IL-4 (human) | ATGGGTCTCACCTCCCAACT | GATGTCTGTTACGGTCAACTCG |
| IL-6 (human) | ACTCACCTCTTCAGAACGAATTG | CCATCTTTGGAAGGTTCAGGTTG |
| IL-10 (human) | GACTTTAAGGGTTACCTGGGTTG | TCACATGCGCCTTGATGTCTG |
| IL-34 (human) | AAACAAAGCTCCGTCCTAAACTG | GCCGCATACTGCAATGAGG |
| CCL-2 (human) | CAGCCAGATGCAATCAATGCC | TGGAATCCTGAACCCACTTCT |
| CCL-22 (human) | ATCGCCTACAGACTGCACTC | GACGGTAACGGACGTAATCAC |
| TNF-α (human) | CCTCTCTCTAATCAGCCCTCTG | GAGGACCTGGGAGTAGATGAG |
| CSF-1 (human) | TGGCGAGCAGGAGTATCAC | AGGTCTCCATCTGACTGTCAAT |
| CSF-1 (mouse) | GTGTCAGAACACTGTAGCCAC | TCAAAGGCAATCTGGCATGAAG |
| ARG1 (human) | GTGGAAACTTGCATGGACAAC | AATCCTGGCACATCGGGAATC |
| CD86 (human) | CTGCTCATCTATACACGGTTACC | GGAAACGTCGTACAGTTCTGTG |
| CCR7 (human) | TGAGGTCACGGACGATTACAT | GTAGGCCCACGAAACAAATGAT |
| GAPDH (human) | GGAGCGAGATCCCTCCAAAAT | GGCTGTTGTCATACTTCTCATGG |
| GAPDH (mouse) | TCAAAGGCAATCTGGCATGAAG | GAGTTGCTGTTGAAGTCGCA |
| F4/80 (mouse) | CTCAGTCTGCACCAATATCCTG | CCACAGAGTTAGAGCAGTTGGAA |
| iNOS (mouse) | ACATCGACCCGTCCACAGTAT | CAGAGGGGTAGGCTTGTCTC |

**Supplementary Table 2 – antibodies used in this study**

| **Vendor** | **Antibody** | **Clone type** | **Host species** | **Catalog number** | **Use** | **Working dilution** |
| --- | --- | --- | --- | --- | --- | --- |
| Santa Cruz | Anti-AR | Polyclonal IgG | Rabbit | sc-816 | WB/IHC/ChIP | 1:1000/1:100/1:100 |
|  | Anti-GAPDH | Monoclonal IgG1 | Mouse | sc-32233 | WB | 1:1000 |
|  | Anti-CSF-1 | Polyclonal IgG | Goat | sc-1324 | WB | 1:1000 |
| Abcam | Anti-CSF-1 | Polyclonal IgG | Rabbit | ab99178 | IHC | 1:100 |
|  | Anti-CD163 | Monoclonal IgG | Rabbit | ab182422 | IHC | 1:100 |
|  | Anti-CD206 | Polyclonal IgG | Rabbit | ab64693 | IHC | 1:200 |
|  | Anti-F4/80 | Polyclonal IgG | Rabbit | ab100790 | IHC | 1:100 |
|  | Anti-CD68 | Polyclonal IgG | Rabbit | ab125212 | IHC | 1:100 |
|  | Anti-iNOS | Polyclonal IgG | Rabbit | ab15323 | IHC | 1:100 |
| Bio-Rad | Anti-CD206 | Monoclonal IgG | Mouse | MCA2235 | IF | 1:100 |
| Boster | Anti-αSMP | Monoclonal IgG2a | Mouse | BM0002 | IHC | 1:100 |
| eBioscience™ | Anti-CD206 | Monoclonal IgG1 | Mouse | 12-2069-42 | FC | 5ul/test |
| eBioscience™ | kappa Isotype Control, PE | IgG1 | Mouse | 12-4714-81 | FC | 1:50 |
| BioLegend | kappa Isotype Control, FITC | IgG1 | Mouse | 400107 | FC | 1:50 |
| BioLegend | Anti-CD163 | Monoclonal IgG1 | Mouse | 333618 | FC | 5ul/test |

WB, western blot; IHC, immunohistochemistry; IF, immunofluorescence staining; ChIP, chromatin immunoprecipitation; FC, flow cytometric analysis.

**Supplementary Table 3** - Sequences for primers targeting miR-185-5p promoter region in ChIP-qPCR assay

| **Bind site** | **Forward primer (5` to 3`)** | **Reverse primer (5` to 3`)** |
| --- | --- | --- |
| I | CCATGTGCCTGTGTCATGC | ATCTGCTGATCCCCGCCA |
| II | GCATGGCTTTGACCACTCTG | CTACAGGAGGGTTGGTGTCC |
| III | TGCGCCCAGATCAAGATATG | GACCTGTGACCTTGCCTTTG |
