# Supplementary material for: Loss of the androgen receptor suppresses intrarenal calcium oxalate crystals deposition via altering macrophage recruitment/M2 polarization with change of the miR-185-5p/CSF-1 signals
Source: Cell Death Dis. 2019 Mar 20;10(4):275. doi: 10.1038/s41419-019-1358-y (PMC6427030; doi:10.1038/s41419-019-1358-y)
Supplement: Supplementary file 2 — Supplementary figure legends [file 41419_2019_1358_MOESM2_ESM.docx]

**Supplementary figure legends**

**Figure 1S. (A)** Experimental outline for macrophage migration assay and crystals phagocytosis assay. The CM were collected from M-1 cells (scr/shAR) treated with 20 μg/cm^2^ COM for 24 hrs. For macrophage migration assay, 1×10^5^/well RAW264.7 cells were added in the upper chambers, and the CM were placed into the lower chambers of transwell plates. For crystals phagocytosis assay, the RAW264.7 cells were incubated in CM that was diluted with 10% heat-inactivated serum DMEM media at 1:1 for 3 days. **(B)** The RAW264.7 cells migration to the M-1 (scr/shAR) CM are shown. **(C)** CM from AR-depleted M-1 cells showed increased mRNA levels of markers of M2 phenotypic MΦs, including CD163, CD206, ARG-1, IL-10 and IL-4 in RAW264.7 cells after 72 hrs of incubation compared to CM from control (scr) M-1 cells. **(D)** Analysis of the COM crystals phagocytosis ability of RAW264.7 cells after incubation with M-1 (scr/shAR) CM for 72 hrs, then RAW264.7 cells were treated with 15 μg/cm^2^ Ponceau-S-stained COM crystals. At 24 hrs the crystals cells uptake was evaluated by optical microscopy. Representative images show light microscopy photographs taken 24 hrs after adding crystals (left panel). Quantification shows mean ± SD percent of MΦs containing phagocytized red material (right panel). **(E)** The qRT-PCR analysis of CSF-1 expression in M-1 cells with/without knock down of AR. Cells were pre-treated with 20 μg/cm^2^ COM crystals for 24 hrs. **(F)** The level of CSF-1 in the CM of M-1 cells was detected by ELISA. **(G)** Knocking down CSF-1 interrupted the AR knockdown-mediated increase of RAW264.7 cells migration. **(H)** CM from AR-depleted M-1 cells led to increase mRNA levels of markers of M2 phenotypic MΦs (CD206, CD163 and Arg-1), while knocking down CSF-1 in M-1 cells interrupted AR knockdown-mediated M2-MΦs markers change. **(I)** Knocking down CSF-1 in M-1 partly reversed the AR knockdown-enhanced COM crystals phagocytic ability of RAW264.7. **(J)** qRT-PCR analysis of miR-185-5p in M-1 cells with shAR compared to control. **(K)** The protein levels of CSF-1 in M-1 CM were determined by ELISA after co-transfection with shAR and miR-185-5p (vs. scramble). **(L)** RAW264.7 cells migration to the CM from M-1 cells with four groups (scramble, shAR, miR-185-5p, and shAR + miR-185-5p). **(M)** CM from AR-depleted M-1 cells led to increase mRNA levels of markers of M2 phenotypic MΦs (CD163 and CD206), while knocking down CSF-1 in M-1 cells interrupted AR knockdown-mediated M2-MΦs markers change. **(N)** Overexpressing miR-185-5p in M-1 cells partly reversed the AR knockdown-enhanced COM crystals phagocytic ability of RAW264.7. For **B, D, G, I, L,** and **N,** quantitations are at the right and all quantitations are mean ± SD, *P<0.05, ** P<0.01, *** P < 0.001.

**Figure 2S. (A)** The second shAR targeted sequence. **(B)** qRT-PCR (upper panels) and western blot (bottom panels) show the AR knockdown efficiency of the second shAR in HK-2 and HK-8 cells. **(C)** Macrophage migration to the RTCs CM. The M0-MΦs migration to the HK-2 [scramble/shAR(2)] CM and to the HK-8 [scramble/shAR(2)] CM were shown. **(D)** Analysis of the COM crystals phagocytosis ability of MΦs after incubation with HK-2 [scramble/shAR(2)] CM or [scramble/shAR(2)] CM for 72 hrs. After 3-days incubation with CMs, the MΦs were treated with 15 μg/cm^2^ Ponceau-S-stained COM crystals. At 24 hrs the crystals MΦs uptake was evaluated by optical microscopy. Representative images show light microscopy photographs taken 24 hrs after adding crystals (left panel). Quantification shows mean ± SD percent of MΦs containing phagocytized red material (right panel). **(E)** Knocking down CSF-1 with second shRNA interrupted the AR knockdown-mediated increase of M0-MΦs migration. **(F)** Knocking down CSF-1 with second shRNA in HK-2 and HK-8 partly reversed the AR knockdown-enhanced COM crystals phagocytic ability of MΦs. For **C-F,** quantitations are at the right and all quantitations are mean ± SD, * P<0.05, ** P<0.01.
