# Supplementary figures and images for: Loss of the androgen receptor suppresses intrarenal calcium oxalate crystals deposition via altering macrophage recruitment/M2 polarization with change of the miR-185-5p/CSF-1 signals
Source: Cell Death Dis. 2019 Mar 20;10(4):275. doi: 10.1038/s41419-019-1358-y (PMC6427030; doi:10.1038/s41419-019-1358-y)

# Supplementary Figure 1

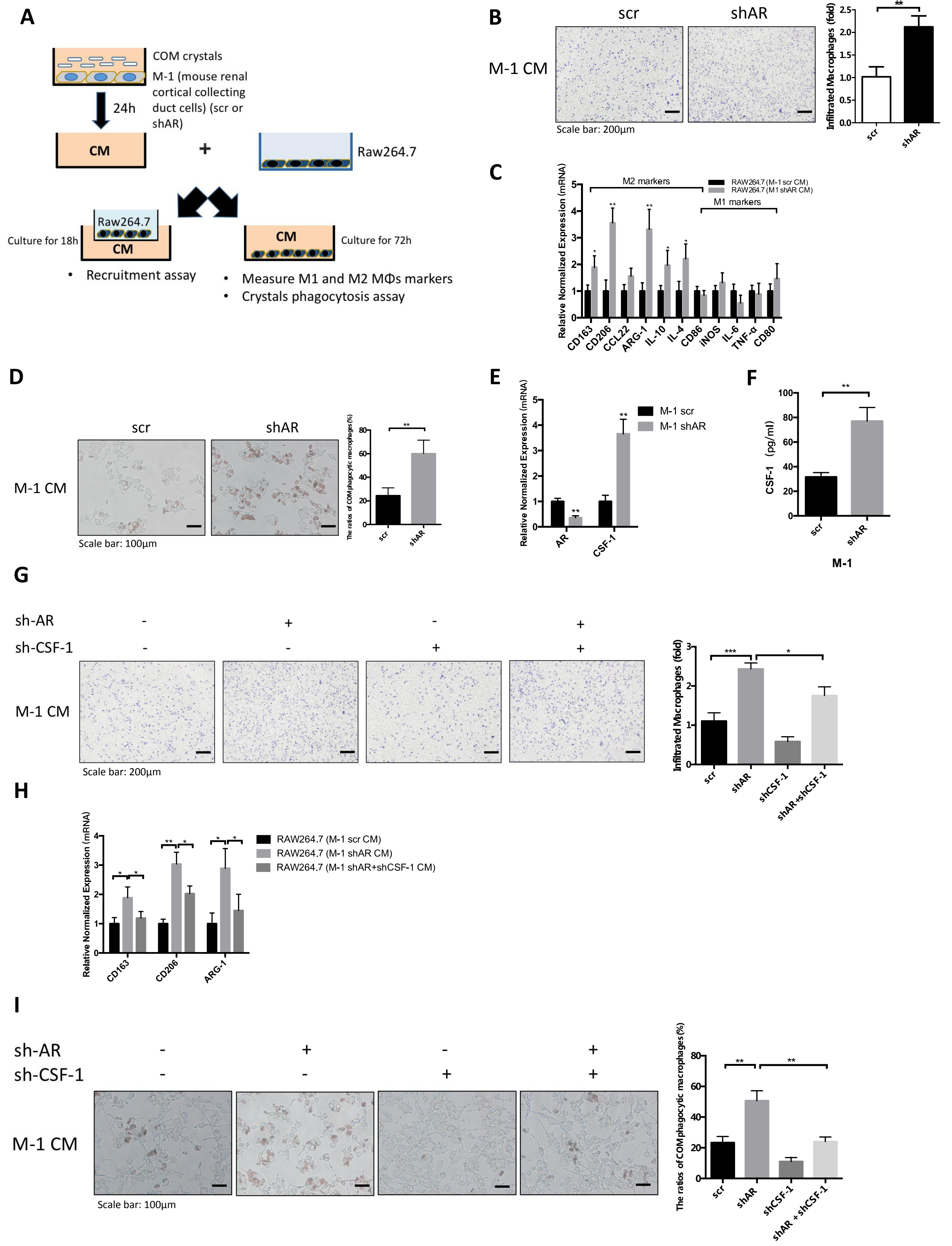

Supplement: Supplementary file 3 — Supplementary Fig 1-1 [file 41419_2019_1358_MOESM3_ESM.pdf]

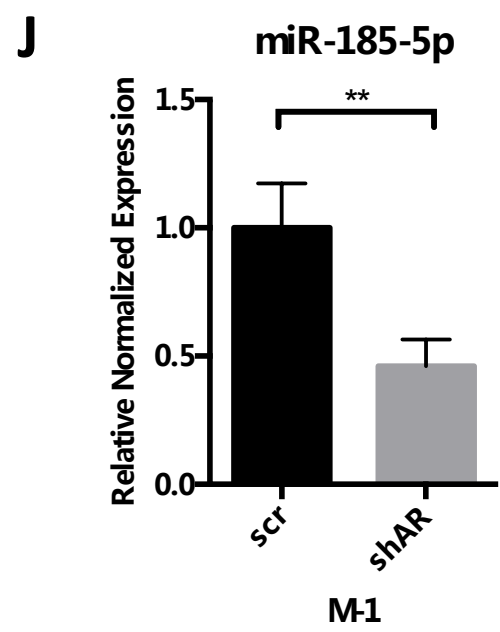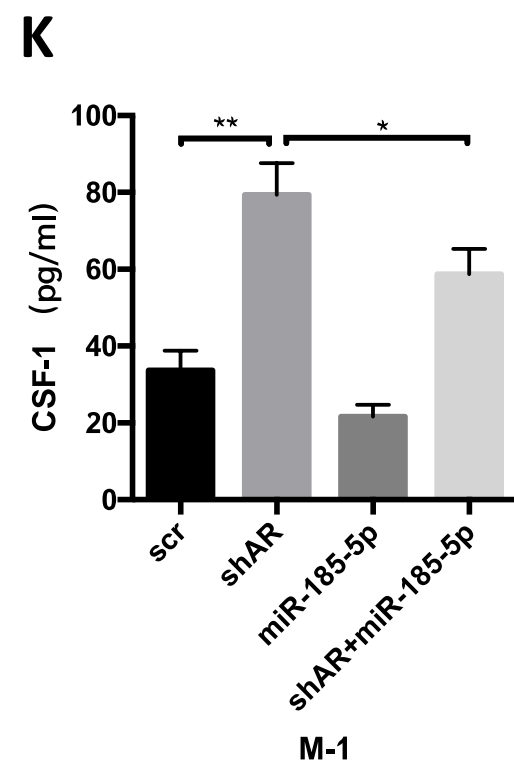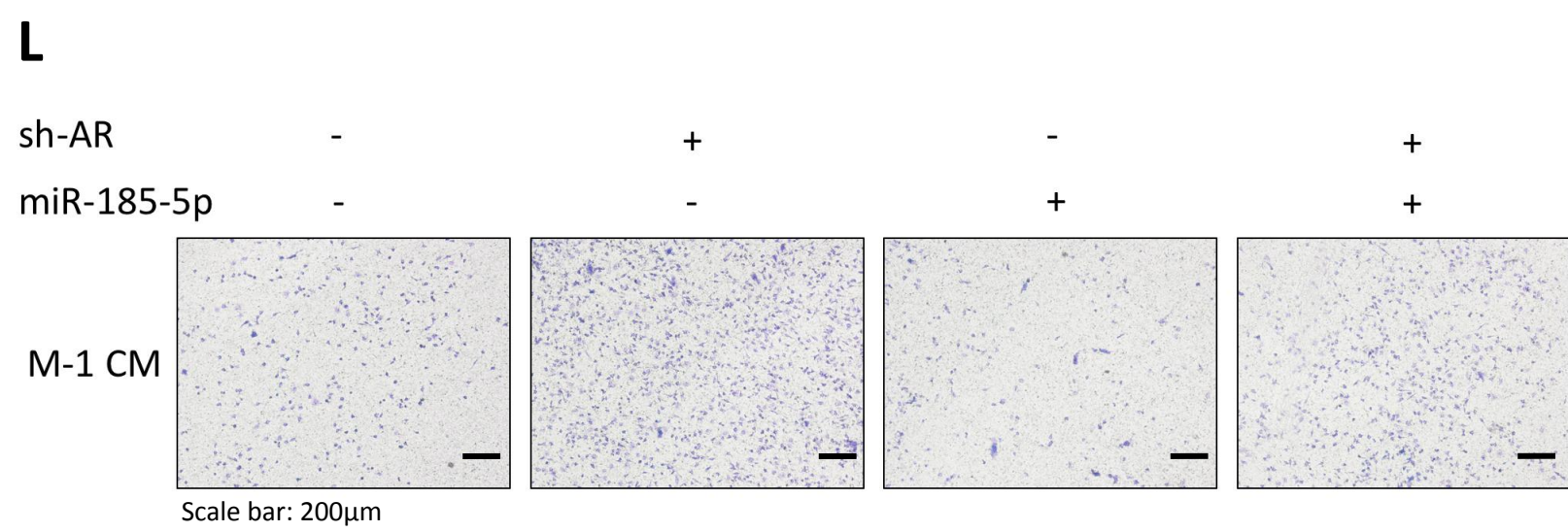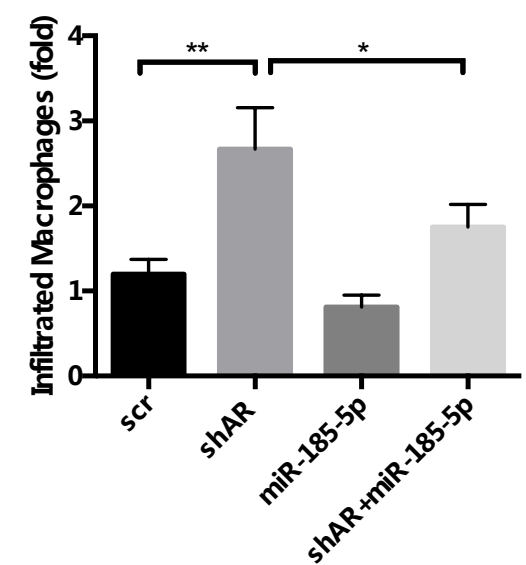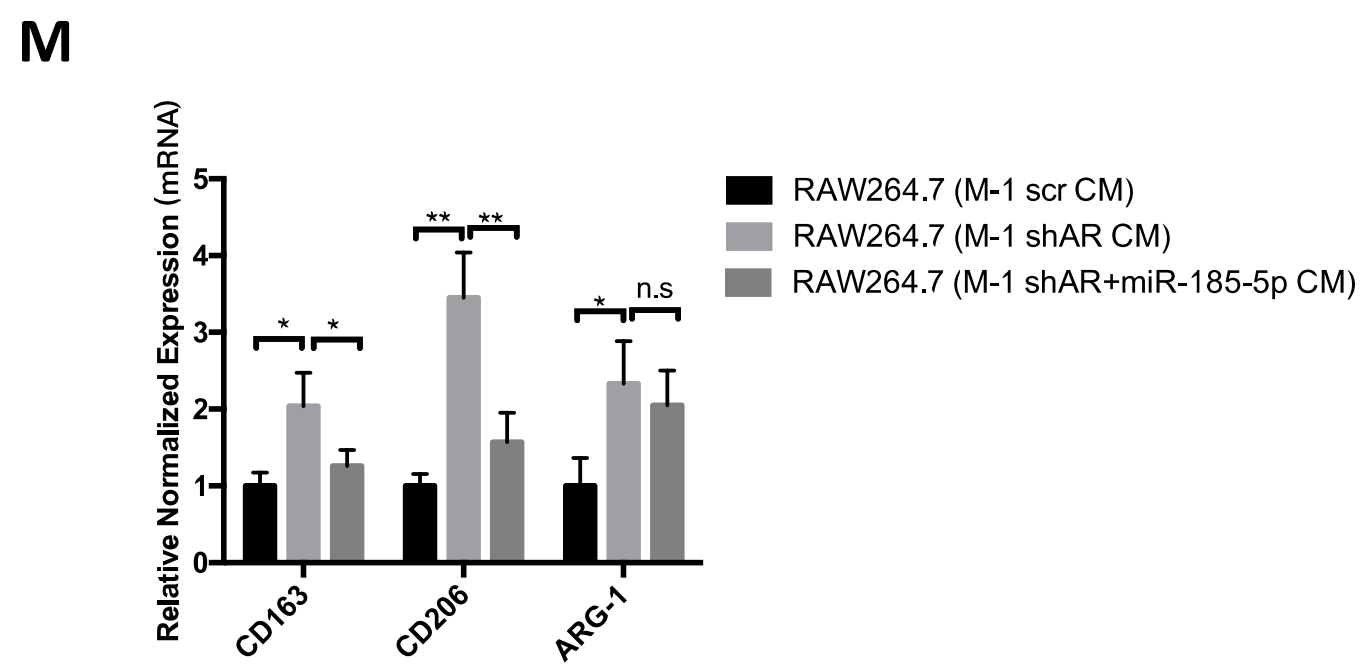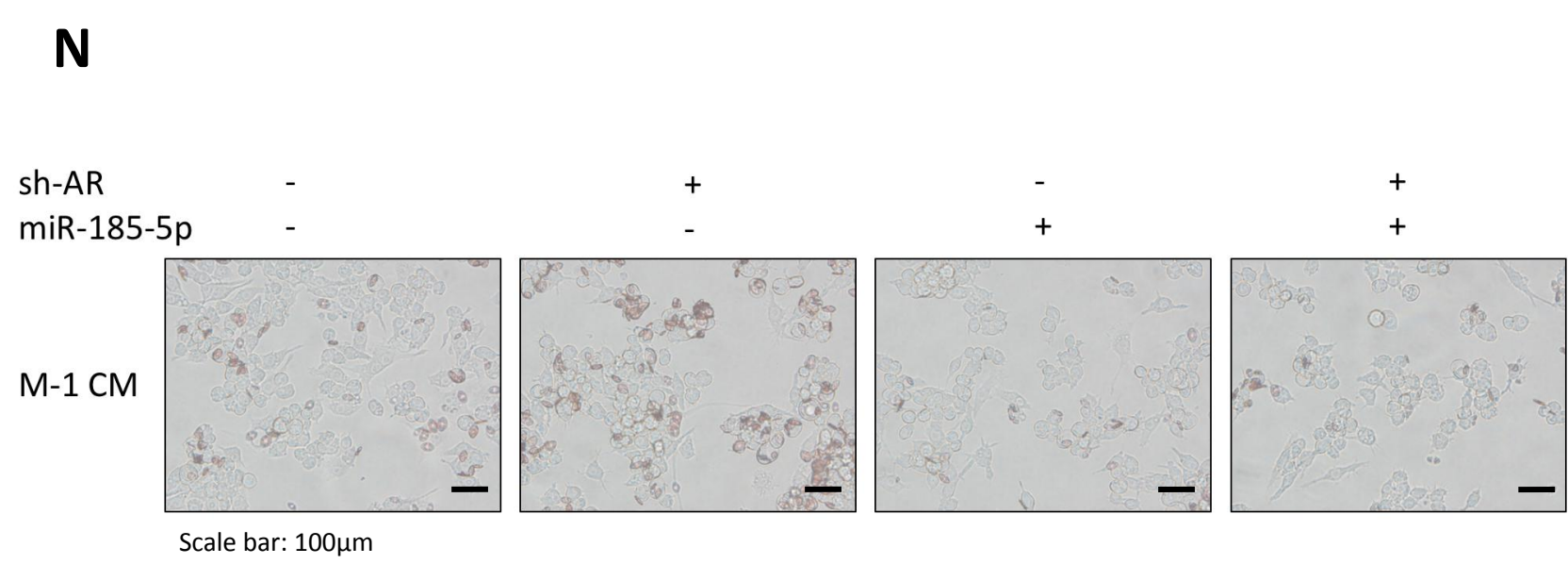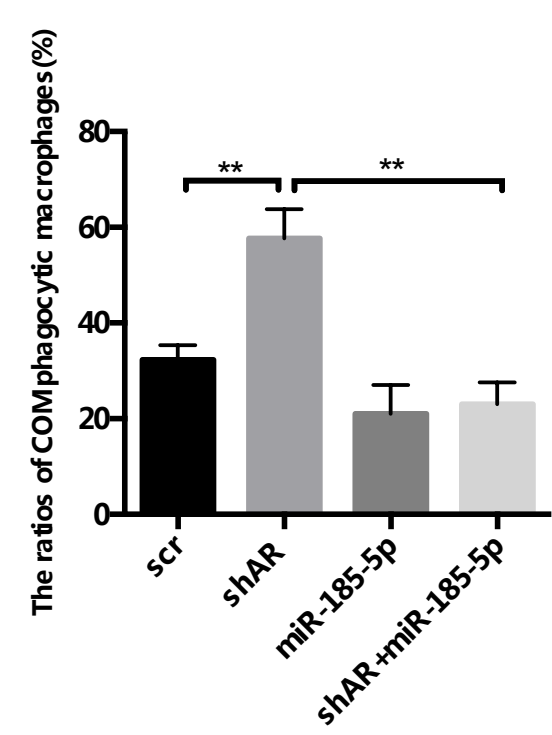

Supplement: Supplementary file 4 — Supplementary Fig 1 continues [file 41419_2019_1358_MOESM4_ESM.pdf]

# Supplementary Figure 2

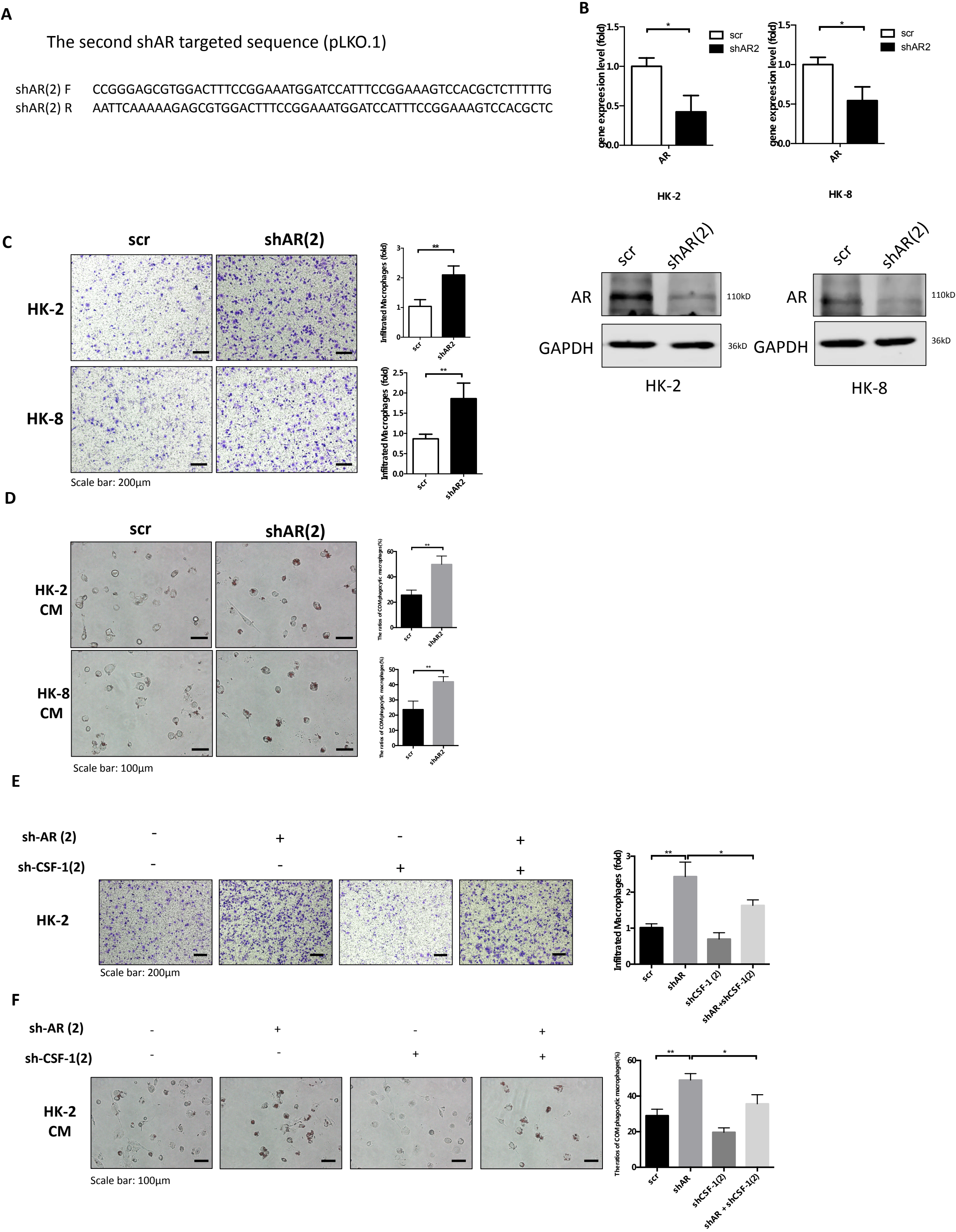

Supplement: Supplementary file 5 — Supplemental Figure 2 [file 41419_2019_1358_MOESM5_ESM.pdf]
